# Aggravation of reactive nitrogen flow driven by human production and consumption in Guangzhou City China

**DOI:** 10.1038/s41467-020-14699-x

**Published:** 2020-03-05

**Authors:** Yue Dong, Linyu Xu, Zhifeng Yang, Hanzhong Zheng, Lei Chen

**Affiliations:** State Key Joint Laboratory of Environmental Simulation and Pollution Control, School of Environment, Beijing Normal University, No. 19, Xinjiekouwai Street, Haidian District Beijing, 100875 China

**Keywords:** Element cycles, Urban ecology

## Abstract

Human activities reshape the global nitrogen (N) cycle and affect environment and human health through reactive nitrogen (Nr) loss during production and consumption. In urbanized regions, the N cycle is greatly mediated by complex interactions between human and natural factors. However, the variations in sources, magnitude, spatiotemporal patterns and drivers of Nr flows remain unclear. Here we show by model simulations, anthropogenic perturbations not only intensify Nr input to sustain increasing demands for production and consumption in Guangzhou city, China, but also greatly change the Nr distribution pattern in the urban system, showing a substantial Nr enrichment in the atmosphere and a relatively low retention capacity of Nr in the terrestrial system. Our results highlight the strong anthropogenic effect of urban systems on the N cycle to suggest sustainable human activity changes to harmonize the relationship between Nr behaviors and human drivers.

## Abstract (web summary)

There lacks research to figure out the variations in sources, magnitude, and spatiotemporal patterns of Nr flows in urban system. Here the authors develop a coupled human-natural urban nitrogen flow analysis model and show that anthropogenic perturbations not only intensify Nr input to sustain increasing demands for production and consumption of cities, but also greatly change the Nr distribution pattern in the urban system.

## Introduction

Nitrogen (N) is an essential element for all biological tissues, and the N cycle is one of the main biogeochemical cycles that can have a substantial and wide-ranging impact on the ecological environment^1–3^. Human activities have greatly changed the global N cycle and increased the creation of reactive nitrogen (Nr) since Haber and Bosch succeeded in artificially synthesizing ammonia^4,5^. Influenced by strong anthropogenic effects, urban systems (especially megalopolises) have played a significant role in the tremendous changes in Nr creation and the N cycle^6^. A substantial body of research has shown that the amount of circulating Nr in cities is several times higher than that in surrounding non-urban ecosystems^7,8^. The relatively high demand for food, industrial goods, and energy due to urban life has driven high-throughput food N production in rural areas, led to massive industrial N fixation, and caused the release of organic N from fossil fuels. However, only a small proportion of anthropogenic Nr can be used efficiently by humans, and most Nr is lost or accumulated in the environment^9^. The intensification of Nr release into the environment has had negative effects on both the ecosystem and human health^10^, such as stratospheric ozone depletion^11^, acid rain^12^, water eutrophication^11,12^, biodiversity loss^13^, and human heath impairment^11^.

The former N budget has been studied at the global^14^, national^15^ and regional^16,17^ levels. Compared with large scales, in urban regions, the N cycle is greatly mediated by complex interactions between human and natural factors, resulting in variations in sources, magnitude and spatiotemporal patterns^18,19^. Some urban-scale studies have mainly focused on specific aspects of the N balance in natural and socioeconomic systems, such as the cycling process of nutrients between urban areas and farmland^20^, N flow in urban food consumption^21,22^ and N metabolism network flow analysis^23^. However, a detailed and holistic approach to mapping the coupled human-natural urban N flow trajectory is still lacking. Notably, some important urban N flow fates driven by human production and consumption activities are not fully understood. For example, figuring out the fate of urban industrial N has been challenging. Research has shown that the fate of 26 Tg N y^−1^ retained worldwide remains unknown, though it is most likely related to the cycling process of industrial N^12^. Synthetic industrial N is difficult to decompose and tends to accumulate in human settlements^4,14,24^, thereby changing the pattern of Nr metabolism in modern cities, which may have a significant impact on the environment and human health. Moreover, further quantitative analysis of the driving effect of human activities on Nr creation in urban systems are needed. Studies investigating the impact of human interference on the N cycle at the national level or watershed scale could ignore some sudden hot spots and new pollution sources^15,25^, such as the recent rapid growth of industrial N emissions in urban systems^26^, which could amount to a missed opportunity to develop abatement management strategies targeting these sources of pollution.

The primary purposes of this study are to develop a new coupled human-natural nitrogen flow analysis model at the urban scale and then use this model to investigate how the N flow originated, modified, and affected the environment in a typical megalopolis. The model core is based on the human activities of the N cycle and associated effect on various subsystems. The city of Guangzhou (22°26′–23°56′N, 112°57′–114°03′E) was selected as the study area as it is one of the most dynamic economic cities in China and the Asia Pacific region and has a dense population with an intense level of human activity. Guangzhou is also an advanced manufacturing base and a modern service base with a global influence. Guangzhou spans 7434 km^2^ and, in 2015, had a population of 1.35 × 10^7^ and an average urbanization rate of 85.53%^27^. The gross domestic product (GDP) of this city was 1,810.04 billion yuan, which was divided among primary, secondary, and tertiary industries, accounting for 1.25, 31.64, and 67.11% of this amount, respectively^27^. The concentrated population, rapidly developed industry and massive fossil fuel combustion have all accelerated Nr creation and caused N pollution in water sources and the atmospheric environment^28^.

Based on the coupled human-natural N flow analysis model, we analyse the temporal (1995–2015) variations of the N balance with respect to sources, fluxes, and fates with uncertainty in Guangzhou. Specifically, we carry out a life cycle analysis of food N and industrial N, which are dominated by human production and consumption, and the impacts of the N flow process on the environment are highlighted. We find that Nr input into the urban system is manifested in not only artificial intensification but also the change of the input structure. The massive dependence of the urban system on external agricultural N-contained products results in more N pollution into surface water from consumption rather than production. The Haber-Bosch N fixation tends to generate industrial synthetic products for human consumption rather than fertilizers for agricultural use, thus leading to the durative accumulation of industrial Nr in human settlements. Anthropogenic perturbations greatly change the Nr distribution pattern in the urban system, showing a substantial Nr enrichment in the atmosphere and a relatively low retention capacity of Nr in the terrestrial system. Subsequently, using an extended Stochastic Impacts by Regression on Population, Affluence, and Technology (STIRPAT) model, we quantitatively assessed the impact of human activities on Nr creation in the urban system and identified strategies that can be implemented to maintain sustainable development. In the urban system, practical strategies to regulate Nr creation should be focused on reducing the negative effects of industrial Nr, encouraging a reasonable and balanced dietary structure and reducing N loss during energy consumption.

## Results

### Temporal variation of the N balance

Based on a coupled human-natural urban N flow analysis, we found that Nr flows in a typical megalopolis have been artificially intensified to sustain the increasing demands for production and consumption with continuous Nr loss in the environment (Fig. 1). The detailed N fluxes among 12 subsystems can be found in Supplementary Figs. 1, 3–14.

**Fig. 1:**
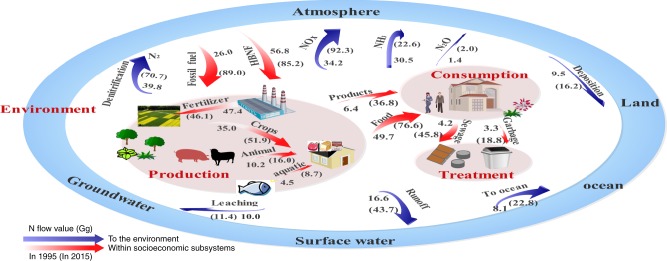
Coupled human-natural urban nitrogen flow analysis in Guangzhou from 1995 to 2015. N flows among production, consumption, treatment and environment process groups under anthropogenic perturbations. The numbers in brackets and out of brackets represent N fluxes in 2015 and 1995, respectively. HBNF, Haber- Bosch N fixation. Units are in Gg N y^−1^.

**Fig. 2:**
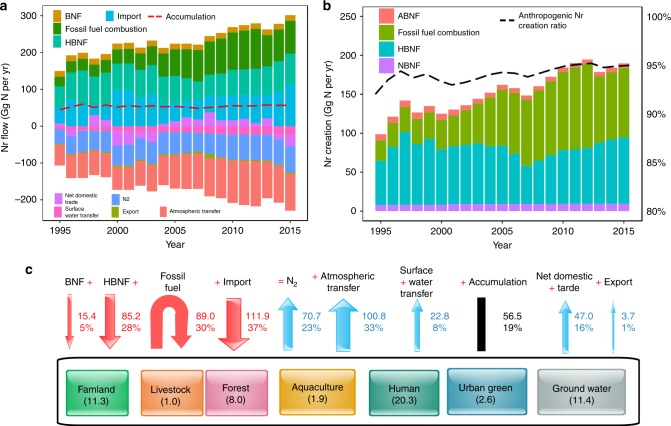
Temporal variation of N balance in Guangzhou (Units are in Gg N y^−1^). **a** The Nr input, output, and accumulation. **b** The Nr creation. **c** Schematic of N balance in 2015. ABNF, agricultural biological N fixation; HBNF, Haber- Bosch N fixation; NBNF, natural biological N fixation; Atmospheric transfer: Nr transfer to the surrounding areas; Surface water transfer: Nr transfer to oceans.

Over the past two decades, the total annual Nr inputs in Guangzhou increased from 142.5 Gg to 301.5 Gg and showed slight fluctuation (Fig. 2a). Anthropogenic Nr creation (excluding natural BNF) continued to increase from 90.9 Gg in 1995 and reached 180.0 Gg in 2015, contributing to 95.0% of the total Nr creation (BNF, HBNF, and N fixation during fossil fuel combustion) (Fig. 2b). The anthropogenic dominating inputs included trade imports, N fixation during fossil fuel combustion, and HBNF, which accounted for 37.1, 29.5, and 28.3%, respectively. Fossil fuel combustion increased from 26.0 Gg to 89.0 Gg due to the growing demand for industrial production and household utilization in the urban system. The input intensity of Nr fixation from fossil fuel combustion in Guangzhou in 2015 was 119.7 kg N ha^−1^ y^−1^, which was much greater than the value of all of China (8.85 kg N ha^−1^ y^−1^)^15^. HBNF was used to produce N fertilizers and other synthetic ammonia products (e.g., plastics, synthetic rubbers, synthetic fibers, detergents, drugs and other products). Our estimate of the HBNF input intensity in 2015 was 114.6 kg N ha^−1^ y^−1^, which was higher than the average value in China (~48.9 kg N ha^−1^ y^−1^)^29^. Another important characteristic of Nr inputs in Guangzhou was the heavy reliance on external supplies, especially agricultural products.

The total N outputs were 99.2 Gg in 1995 and 245.0 Gg in 2015, and approximately 50.4% was Nr that was ultimately discharged into the environment in 2015 (Fig. 2a, c). This large flux of Nr emissions could lead to serious atmospheric and hydrosphere problems. As a result of Nr inputs and outputs in the coupled human-natural urban system, the Nr accumulations were 44.7 Gg in 1995 and 56.5 Gg in 2015. Human and farmland subsystems contributed to the greatest successive accumulation (Fig. 2a). In 2015, human subsystem contributed to 35.9% of the total accumulation, followed by farmland (20.0%), forest (14.2%), groundwater (20.2%), urban green (4.6%), aquaculture (3.4%), and livestock (1.7%) (Fig. 2c). Over the past two decades, Guangzhou gradually transformed into a typical advanced manufacturing and modern service-based city driven by substantial human consumption. Industrial synthetic products for human consumption resulted in an accumulation due to their long service lives. In contrast, in the entire country, farmland largely contributed to the Nr accumulation due to massive chemical fertilizer input and large cultivation scale^24^.

To ensure the robustness of the N flow results, we conducted a Monte Carlo simulation to test the propagation of input uncertainties into the successive and final results. The uncertainty results of key N flows in Fig. 2c are shown in Table 1, and the mean, 5^th^ and 95^th^ percentiles are provided. Small uncertainty was observed in the N flows derived from the official statistics. For example, 89.0 Gg (66.7 Gg, 113.0 Gg) N fixation from fossil fuel combustion in 2015 with range of ~25% was estimated by consumption of all types of fossil energy and corresponding NOx emission factors. Nr accumulation was calculated as the difference between inputs and outputs, and all uncertainties in the inputs and outputs may be additive in Nr accumulation, e.g., Nr accumulation in the farmland subsystem was 11.3 Gg (2.1 Gg, 22.5 Gg) with range of ~90%. Besides, we provided uncertainty analysis of partial N flows due to activities data and parameters respectively in Supplementary Fig. 15. Uncertainties due to parameters contributed more than that due to activity data to the full uncertainties. Moreover, we compared partial N flows with previous estimates from China and other cities to validate the rationality of the calculation results, which is provided in Supplementary Table 22 and Supplementary Discussion 2.

**Table 1:** Key N flows with uncertainty ranges in Guangzhou in 2015 (Gg N y^−1^).

N balance	Range		
	Mean	5th	95th
**Inputs**			
BNF	15.4	12.6	18.3
HBNF	85.2	75.6	95.3
Fossil fuel	89.0	66.7	113.0
Import	111.9	88.3	135.2
**Outputs**			
N_2_	70.7	52.8	87.4
Atmospheric transfer	100.8	76.5	123.9
Surface water transfer	22.8	11.1	34.6
Net domestic trade	47.0	23.2	70.3
Export	3.7	3.0	4.4
**Accumulations**			
Farmland	11.3	2.1	22.5
Livestock	1.0	0.8	1.3
Forest	8.0	5.3	10.6
Aquaculture	1.9	0.1	4.1
Human	20.3	10.5	29.4
Urban green	2.6	1.7	3.5
Ground water	11.4	8.9	16.8

### Industrial Nr fate analysis

**Fig. 3:**
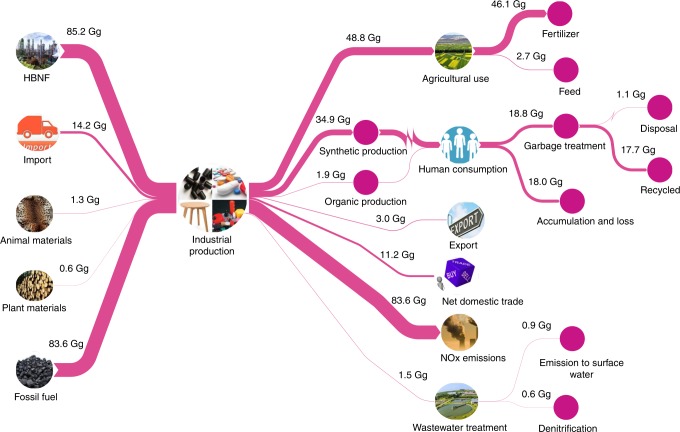
Life cycle analysis of the industrial Nr fate of Guangzhou in 2015. Anthropogenic inputs of Nr through HBNF, import, agriculture materials and fossil fuel provide industrial products (including synthetic and organic products) to meet human demands, a fraction of Nr is for agriculture use and human consumption, the rest is transferred outside the system or lost to the environment. HBNF, Haber-Bosch N fixation. Units are in Gg N y^−1^.

Increased anthropogenic inputs of Nr provide more industrial products (including synthetic and organic products) to meet human demands. During production and consumption processes, a substantial proportion of Nr is lost to the environment. The synthetic products are mainly used as N fertilizer and feed for agricultural purposes and synthetic ammonia products (e.g., plastics, synthetic rubbers, synthetic fibers, detergents, drugs and other products) to meet human consumption demands. Synthetic industrial Nr is generally found near human settlements. Environmental and health problems driven by industrial Nr have become increasingly serious^4,10^. Here, we performed a life cycle analysis of industrial N to obtain a more holistic understanding of N fate from production to consumption in the urban system (Fig. 3).

The Nr flux in the industrial subsystem sharply increased from 125.2 Gg in 1995 to 184.9 Gg in 2015 primarily due to an increase in HBNF and N fixation through fossil fuel combustion. In 2015, 46% of Nr inputs into industrial production were used to support agricultural activities and human consumption, while residual Nr was discharged into the environment or transferred to the external system. The fossil energy input into the industrial system was substantial, resulting in the release of 83.6 Gg of NOx emissions into the atmosphere in 2015 mainly caused by emissions from automobiles, aircraft, internal combustion engines, and industrial furnaces. In Guangzhou, the NO_2_ concentration (47 μg m^−3^) exceeded the acceptable maximum rate outlined by the second grade of the Ambient Air Quality Standards in China (40 μg m^−3^) in 2015^28^, leading to negative environment and human health effects. Therefore, controlling industrial NOx emissions is a vital and urgent issue in Guangzhou.

HBNF is one of the most important new Nr inputs into circulation in Guangzhou. HBNF increased from 56.8 Gg in 1995 to 85.2 Gg in 2015. In 1995, small quantities of HBNF entered the industrial subsystem to provide synthetic ammonia products for human consumption; the most important output from HBNF was fertilizer N (i.e., 52.3 Gg, 92% of HBNF), most of which was designated for farmland use, which is very similar to the HBNF fate observed in China in 2010 (i.e., fertilizer N accounted for 87% of HBNF)^24^. However, in 2015, synthetic ammonia products accounted for ~41% of the HBNF input in Guangzhou, while the remaining ~57% was associated with agricultural use, leading to the accumulation of synthetic ammonia products in the human subsystem. The total accumulation of synthetic ammonia products in the human system reached 18.0 Gg in 2015, which was approximately six times higher than that in 1995 (3.1 Gg). The load of garbage treatment (mainly from synthetic ammonia waste) increased from 3.3 Gg in 1995 to 18.8 Gg in 2015, most of which was recycled to the human subsystem.

### Food Nr fate analysis

**Fig. 4:**
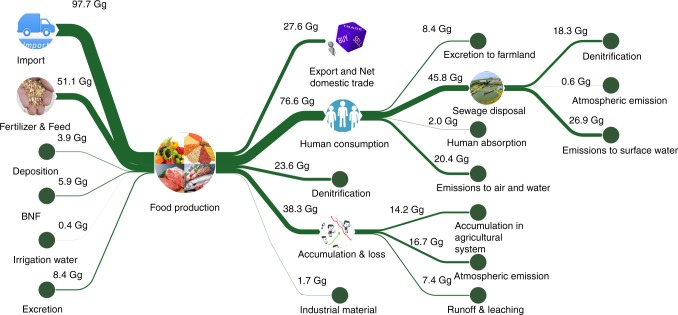
Life cycle analysis of the food Nr fate of Guangzhou in 2015. Inputs of Nr to food production include import, BNF, organic and chemical fertilizer, N deposition, feed, and irrigation water, a fraction of Nr is for human food consumption, the rest is transferred outside the system or lost to the environment. BNF, biological N fixation; Units are in Gg N y^−1^.

Figure 4 shows the life cycle analysis of food Nr fate from production and consumption in Guangzhou. Inputs of Nr to food production (i.e., including crops, livestock, and aquaculture products) increased from 100.1 Gg to 167.8 Gg from 1995 to 2015 mainly due to the growth of import products. Correspondingly, Nr inputs from fertilizer and ammoniated feed slightly declined from 52.3 Gg to 51.5 Gg during this 20-year period. To obtain food, a developed city such as Guangzhou relies on external input more than local production, resulting in relatively low Nr emissions and accumulations from agricultural production in the environment. In 2015, a total of 38.3 Gg of Nr from food production was transferred to the environment, resulting in accumulations in the agricultural system (14.2 Gg), emission to the atmosphere (16.7 Gg), and discharge to the water (7.4 Gg). In contrast to production, consumption by the dense population in the urban system greatly increased Nr emissions in the environment. In 2015, 76.6 Gg food N was input into the human subsystem, resulting in 56.3 Gg released into the environment, 65.0% of which was discharged into surface water bodies. In urban systems, greater attention should be paid to Nr management in food consumption to reduce Nr losses.

Approximately 25% of the Nr input into the farmland was transferred as food to human consumption. The nitrogen use efficiency (i.e., NUE, which is calculated as the Nr contained in harvest crop products divided by the Nr inputs) of the food production system in the farmland was equivalent to that in China^30,31^ but lower than that observed in North America^32^ and Europe^33^, where it was typically above 50%. Moreover, the human absorption ratio of Nr to input declined from 4.4% in 1995 to 2.6% in 2015. Diets changed during the 20-year period, and the ratio of grain protein to animal protein consumed by humans decreased from 2.4 to 2.1.

In China, excreta has been used as fertilizer for centuries^34^. However, the farmland in the urban system is poorly coupled to the livestock and human subsystems, and the overall N recycling ratios of livestock and human excreta declined from 48 and 70% in 1995 to 42 and 11% in 2015, respectively. Previous studies have examined the entirety of China and explored the consequences of an increasing recycling rate of N from livestock and the human subsystem back to farmland, which shows a substantial reduction in Nr creation^24^. Therefore, to increase the recycling of Nr, it is necessary to improve decentralized stockbreeding management and the construction of waste disposal facilities in the urban system. Such improvements could ensure the sustainability of agriculture while promoting human health and the environment.

### Nr environmental load characteristics

**Fig. 5:**
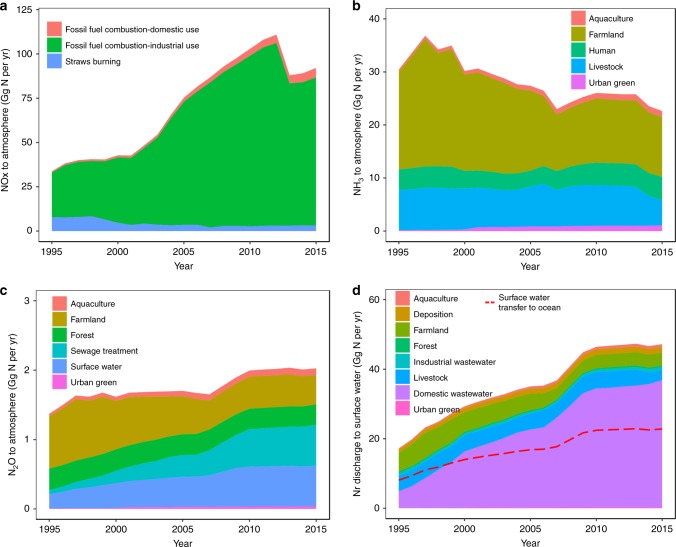
Temporal variation in Nr environmental load characteristics in Guangzhou from 1995 to 2015. **a** NOx emissions to the atmosphere from fossil fuel combustion and straws burning; **b** NH_3_ emissions to the atmosphere from aquaculture, farmland, human, livestock and urban green subsystems; **c** N_2_O emissions to the atmosphere from aquaculture, farmland, forest, sewage disposal, surface water and urban green subsystems; **d** Nr discharge into surface water from aquaculture, atmosphere, farmland, forest, industry, livestock, sewage disposal and urban green subsystems.

During the natural biogeochemical cycling of N, Nr to atmosphere is primarily transformed into stable N_2_ through denitrification^35^. Our analysis demonstrated that in Guangzhou, approximately 23.4% of annual Nr inputs were denitrified as N_2_; the remainder was transferred to the environment and external system or accumulated in the subsystems. During the 20-year period, the increases in Nr emissions in the environment have been durative and extreme ranging from 83.3 Gg in 1995 to 185.9 Gg in 2012 with an annual growth rate of 7.2%. From 2013 to 2015, the rapid upward trend eased with an average level of 163.0 Gg mainly due to lower NOx emissions. In 2013, the Chinese State Council implemented the Air Pollution Prevention and Control Action Plan^36^, which aimed to reduce emissions from power plants, industrial boilers, motor vehicles, and fugitive dust, thus encouraging low NOx emissions. The primary sources of atmospheric emissions were identified as NOx, followed by NH_3_ and N_2_O, which can cause gaseous pollution through atmospheric flow and secondary reactions, such as haze^37^. The change in NOx was the most significant as it was estimated to be 34.2 Gg in 1995 and 92.3 Gg in 2015 (Fig. 5a). After 2002, in particular, the release of NOx, mainly by fossil fuel combustion, increased sharply and suddenly, which is consistent with the increase in energy intensity during this period^38^. The main sources of NOx include fossil fuel combustion from industrial production, which accounted for 73.7% in 1995 and 90.6% in 2015 of the total NOx emission, followed by fossil fuel combustion of domestic consumption and straw burning. NH_3_ volatilization is mainly associated with the use of chemical fertilizers and biological excretion^39^. In Guangzhou, due to the reduction in farmland area, chemical fertilizer use contributed to a gradual decrease in NH_3_ emissions (Fig. 5b). In contrast, the increasingly dense population resulted in a considerable release of NH_3_ into the atmosphere. Moreover, N_2_O from denitrification contributed an increasing amount of Nr in the atmosphere, which is a powerful greenhouse gas^40^, revealing an increase of 1.4 Gg to 2.0 Gg over the past 20 years (Fig. 5c). Previous studies have shown that N_2_O is mainly derived from agricultural soil^41^, and our analysis demonstrated that in an urban system, nitrification and denitrification associated with sewage treatment processes can produce more N_2_O emissions, which accounted for 30.0% of the total N_2_O emissions in 2015.

The Nr input into surface water was 17.2 Gg in 1995 and 47.2 Gg in 2015, with 8.1 Gg and 22.8 Gg transferred to the ocean each year (Fig. 5d). Nr inputs from various sources greatly changed over the past twenty years. These changes were mainly driven by human consumption and discharge, which caused an increase in the fluvial transport of N and reflected an increase from 4.9 Gg to 36.6 Gg in domestic wastewater discharge of N to surface water during the 1995–2015 period. In the near future, with the advancement of urbanization and the improvement in living standards, the discharge of domestic wastewater will continue to increase. In contrast, agricultural Nr emissions into surface water in Guangzhou significantly decreased, and in 2015, Nr transferred to rivers from farmland, livestock, and aquaculture subsystem only accounted for 7.8, 6.1, and 1.7% of the total Nr inputs into rivers.

### Human drivers analysis

An OLS regression estimate of the extended STIRPAT model was used to examine the contributions of four selected individual factors (i.e., population, dietary choice, energy intensity, and industrial level) to anthropogenic Nr creation. The regression coefficients of all explanatory variables were significant, and the R square was 0.940, indicating a good reliability for goodness-of-fit (Table 2, Supplementary Fig. 16 and Supplementary Discussion 1).

**Table 2:** Contributions of socioeconomic factors to changes in anthropogenic Nr creation.

Factors	Annual grow rate(%)	Regression coefficient	Effect coefficient^a^	Contribution rate (%)^b^	*t*-value	VIF
Anthropogenic Nr creation (I)	4.904					
Population (P)	2.352	0.957^***^	1.022	33	4.106	7.614
Diet choice (A)	0.456	2.620^**^	1.012	18	3.061	3.910
Energy intensity (T_1_)	−3.527	0.243^*^	0.991	13	2.502	9.633
Industrial level (T_2_)	0.364	5.617^***^	1.021	30	5.118	2.430
Other factors		−0.274				
R-square	0.940					

Note: Each significance level is associated with a symbol: *p*-values (0.001, 0.01, and 0.05) and corresponding symbols (“***”, “**”, and “*”).

^a^Effect coefficient (effect on anthropogenic Nr creation changes) = (1 + annual grow rate) ^ (regression coefficient).

^b^Contribution rate (contribution to anthropogenic Nr creation changes) = |Effect coefficient − 1|/∑|Effect coefficient − 1| × *R*^2^.

The results showed that population, industrial level, dietary choice, and energy intensity together contributed ~94% to the anthropogenic Nr creation changes in Guangzhou. Over the past two decades, the population in Guangzhou increased by 2.4% per year, resulting in a 2.2% annual increase in anthropogenic Nr creation with a contribution rate of 33%. The population has the greatest potential impact on Nr creation change, which is consistent with the result in China^15^. The industrial level increased by an average of 0.4% per year, was strongly correlated with Nr creation, and showed a 30% contribution rate. The increase in human production and consumption leads to greater levels of Nr creation from HBNF. Sustainable approaches are needed to reduce the negative effects of the increase in industrial Nr products. In Guangzhou, diets have shifted towards a focus on more animal protein, while the N intake from animal food is increasing at an annual rate of 0.46%. Dietary changes resulted in a 1.2% annual increase in anthropogenic Nr creation with a contribution rate of 18%. A reasonable and balanced dietary structure is encouraged. Energy consumption per unit of GDP during this period rapidly declined by 3.53% per year, which negatively contributed to a 13% change in Nr creation. Therefore, promoting efficiency in energy use has a significant effect on the reduction of atmospheric Nr emissions.

## Discussion

Nr input into the urban system is manifested in not only artificial intensification but also the change of the input structure. An important characteristic of Nr input in Guangzhou was heavy reliance on external supplies, especially agricultural products. Consequentially, the substantial increase in Nr creation by N-contained production in other locations was caused by the urban system. The massive dependence of the urban system on external N-contained products resulted in an embodied N pollution transfer from developed consumptive cities to production regions through trade^10^. Moreover, the dependence on external food input resulted in relatively low Nr emissions from agricultural production. In contrast to production, consumption by the dense population in the urban system greatly increased Nr emissions to the environment, especially to the surface water. In 2015, Nr transfer to rivers from domestic wastewater accounted for 78% of the total Nr input into rivers, indicating that in a developed urban system, Nr transfer to rivers is primarily due to human consumption rather than productive and natural processes. The situation differed with respect to the Nr sources of the water in China, where Nr in the water was mainly derived from processes associated with livestock production and the loss of N fertilizer^29^.

In China, HBNF contributed 45.9 Tg N to produce chemical fertilizers in 2010, which is approximately 87% of the total HBNF^15^. However, in Guangzhou, this ratio declined from 92 to 57% during 1995–2015, in contrast, HBNF contributing to other synthetic ammonia products sharply increased from 6 to 41%. HBNF tended to be used to generate synthetic ammonia products for human consumption rather than fertilizers for agricultural use in the urban system, thus resulting in the accumulation of synthetic ammonia products in human settlements. Synthetic ammonia products (e.g., fibers, plastics, rubbers, and dyes) are difficult to decompose, leading to the durative total amount of industrial Nr accumulation being much greater than the annual accumulation, which could contribute to explain the unknown Nr pool worldwide cited by Schlesinger^12^. Long-term cumulative industrial Nr which delays Nr release to the environment could induce the legacy effect of industrial Nr^41^. This type of legacy effects may cause great threats to environmental and human health, for instance, harmful Nr compounds released from paint and dye decomposition^42^.

The fate tracking of Nr introduced into the urban system showed that approximately 23.4 and 16.8% of the annual Nr input was denitrified to N_2_ and outputted through trade, respectively, while the remainder was transferred to the environment or accumulated in the subsystems (Fig. 2). Approximately 33.4% was transferred to the atmospheric environment as NOx, N_2_O and NH_3_, 7.7% was discharged into surface water, and the remaining 18.7% of Nr accumulated in terrestrial systems (including farmland, livestock, forest, aquaculture, human, ground water and urban green subsystems). Differing from our results, a national study showed that the terrestrial system was the main N pool in China, accounting for over two-thirds of the annual N accumulation, followed by water bodies and the atmosphere^15^. Worldwide, in the 1990s, the fate of Nr input was relatively well know; ~18% was transferred to coastal systems via rivers, ~13% underwent atmospheric transfer and was eventually deposited into oceans, and ~22% accumulated in terrestrial systems^3^. Compared with global and national studies, we found that the anthropogenic perturbations greatly changed the Nr distribution pattern in the urban environment, showing substantial Nr enrichment in the atmosphere, resulting in serious atmospheric Nr pollution, especially NOx pollution. In the atmosphere, tropospheric NOx can lead to an increase in the rate of asthma and other respiratory illnesses, particularly in children and other vulnerable populations^43^. Furthermore, analytic results of atmospheric PM2.5 sources have shown that NOx gaseous pollutants in motor vehicle exhausts accounted for a large proportion^44,45^. In contrast, the Nr accumulation in the terrestrial system was much lower than the national level, indicating a reduced capacity to retain Nr input.

Through understanding the socioeconomic driving effect on anthropogenic Nr creation, we focus on sustainable human activity changes to harmonize the relationship between Nr behaviors and human drivers. First, sustainable approaches are needed to reduce the negative effects of industrial Nr. Improving treatment technology could reduce toxic Nr discharge after the production of industrial Nr products. For example, using nano-TiO_2_ photocatalysis to treat dyes could degrade toxic dyes compounds into non-toxic inorganics effectively such as NH_4_^+^-N, NO_3_-N^46^. Besides, increasing reuse rate of newly industrial Nr products could reduce garbage generation, further reduce the negative effect caused by legacy effect^47^, such as garbage siege^48^. Moreover, improving production technology of Nr products could reduce toxic Nr release when they are produced, used or abandoned. For example, using non-diazo-coupling method to synthesize azo dyes (e.g. methyl red) could reduce toxic nitrogenous compounds discharge during the production^49^. Second, a reasonable and balanced dietary structure is encouraged. Compared with grain protein diets, the consumption of animal protein is expected to cause more N losses to the environment^50^. Diet interventions that prevent an increase in the consumption of animal protein could reduce Nr input. Third, reducing N loss during energy consumption is essential. Promoting efficiency in energy use is extremely important to relieve the increase in N fixation during fossil fuel combustion and the emission of NOx^51^. The expanding use of new energy to shift away from fossil fuel consumption is imperative^16^. Given the unique and intense extent of Nr problems in urban systems, these approaches could effectively support the public and decision makers and ultimately yield more sustainable policies.

## Methods

### Dataset

Data were collected from three sources: a number of national, provincial, and urban statistical databases, including the China Statistical Yearbook on Environment (1995–2015), the China City Statistical Yearbook (1995–2015); Guangdong Statistical Yearbook (1995–2015), Guangzhou Statistical Yearbook (1995–2015), Guangzhou Environmental Quality Report (1995–2015); provincial and urban survey and planning documents, such as Guangzhou General Plans for Land Use (2006–2020), and the Guangdong Forest and Grassland Resources Inventory; and existing research results and methodologies.

### Urban nitrogen flow analysis model

In this study, the Nr inputs to the system are a result of N fixation through biology, the Haber-Bosch process, fossil fuel combustion, and imports. In addition, the Nr outputs of the system occur when Nr is reduced to N_2_ or lost to external systems. Within the boundary, we constructed the N flow analysis model (Supplementary Fig. 1a and Supplementary Method 1), which divided the system into four process groups, including production, consumption, treatment, and environment, based on the N fate as modified by human activity. The production group, including farmland, urban green, livestock, forest, aquaculture, and industry subsystems, can process the fixed N input into the food chain, biomass and synthetic products. The consumption group largely focuses on human subsystems. Treatment refers to a group, including sewage and garbage disposal subsystems, that can treat waste Nr and eliminate its negative impact. The environment group includes atmosphere, surface water, and groundwater subsystems. Each subsystem can be viewed as a dynamic unit and a reservoir of Nr. All Nr input from the external system will access one or several subsystems, recycle among the 12 subsystems, or output to the outside.

Based on the principle of the conservation of mass, the N balance^52^ in the whole system and each subsystem is expressed using the following equation:

1 $${\sum} {AC_k} = {\sum} {IN_i} - {\sum} {OUT_j}$$

where *IN*_*i*_ and *OUT*_*j*_ represent the Nr inputs and outputs, respectively; and *AC*_*k*_ represents Nr accumulation.

More precisely, the coupled human-natural urban nitrogen flow analysis model comprises two interrelated models, namely, the Full Nitrogen Flow Analysis (FNFA) model and the Nitrogen Network Calculator (NNC). Based on the substance flow analysis, the FNFA model was developed to characterize the dynamics of the N fate and flux across 12 subsystems (i.e., farmland, urban green, livestock, forest, aquaculture, industry, human, sewage disposal, garbage disposal, surface water, ground water, and atmosphere) throughout the system. The detailed model framework is shown in Supplementary Fig. 1b and Supplementary Method 2. The calculation of input, output and accumulation in each subsystem were described in Supplementary Tables 1–12. Furthermore, the activity data and parameters information in each input, output and accumulation were interpreted in Supplementary Tables 13–21.

The NNC was proposed as a method that could help organize and synthesize large amounts of information with uncertainty using R software when carrying out the N balance calculation^53^. The detailed NNC models are shown in Supplementary Fig. 2 and Supplementary Method 3. By combining the FNFA and NNC models, the N flow analysis of the coupled human-nature urban system automatically produced massive, fast, and synchronous calculations of N fluxes and their interactions.

For uncertainty analysis, we conducted the Monte Carlo simulation to test the propagation of input uncertainties into the successive and final results. The variables were attributed continuous distributions instead of single point estimate. According to the data quality, different continuous distributions were applied and different coefficients of variation (CVs) were set. We classified the CVs of variables into three grades: high, moderate, and low reliabilities, which were assumed to have the values of 0.1, 0.2, and 0.3, respectively^54^. The simulation model ran 10,000 trials by randomly selecting values from the input distributions to generate ranges of outcomes. Besides the single-point estimates, uncertainty analysis of the N flows including means, SDs, CVs, 5th and 95th percentiles were provided. More details of uncertainty analysis are provided in the Supplementary Method 4. Moreover, we compared the partial N flows with previous estimates from China and other cities, which is provided in Supplementary Table 22 and Supplementary Discussion 2.

### Assessment of human driver

The Stochastic Impacts by Regression on Population, Affluence, and Technology (STIRPAT) model was used to assess the contributions of socioeconomic factors to changes in anthropogenic Nr creation. The standard STIRPAT model is facilitated with a logarithm function as a nonlinear transformation function (Eq. (2)). More details of the STIRPAT model are provided in the Supplementary Method 5.

2 $${\mathrm{ln}}I = a + b\,{\mathrm{ln}}\,P + c\,{\mathrm{ln}}\,A + d\,{\mathrm{ln}}\,T + e$$

where, *P*, *A*, and *T* represent population, affluence, and technology. *b*, *c*, and *d* are the coefficients of *P*, *A*, and *T*, respectively; *a* is a constant, and *e* is the error term.

We tested the influence of six related sociological factors (i.e., population, dietary choice, per capita, energy intensity, industrial level, and industrial structure) on anthropogenic Nr creation and used the Pearson’s correlation method to determine the correlations between all factors. Finally, four socioeconomic factors (i.e., population, dietary choice, energy intensity, and industrial level) were selected. Dietary choice was defined as the ratio of animal source food to the total food intake. Energy intensity was defined as the energy consumption per unit of GDP (ton SCE per 10^4^ yuan). The industrial level was defined as the ratio of N_2_ converted to Nr through anthropogenic ammonification to total Nr creation. Dietary choice (A) was used as a proxy for societal affluence. Energy intensity (T_1_) and industrial level (T_2_) were used as proxies for technology. Here, we revised Eq. (2) by adding these factors, thus producing Eq. (3), and an ordinary least squares (OLS) regression was used to evaluate the variables.

3 $${\mathrm{ln}}\,I = a + b\,{\mathrm{ln}}\,P + c\,{\mathrm{ln}}\,A + d_1\,{\mathrm{ln}}\,T_1 + d_2\,{\mathrm{ln}}\,T_2 + e$$

### Reporting summary

Further information on research design is available in the Nature Research Reporting Summary linked to this article.

## Supplementary information

Supplementary Information

Reporting Summary

## Data Availability

All relevant data are publicly available in the supplementary materials and online data repositories, and are available from the authors. The sources of activity data, parameters for N flow analysis are provided in Source Data 1. The source data underlying Figs. 2a–c, 5a–d are provided in Source Data 2.

## Source data

Source Data

## Code availability

Please see supplementary materials for coupled human-natural urban N flow modeling, activity data distribution and parameter distribution in Monte Carlo simulation. Further code details can be obtained from the authors upon request.

## References

[1] Schlesinger, W. H. & Bernhardt, E. S. An analysis of global change. *Biogeochemistry* (2013).

[2] Vitousek PM (1997). Human alteration of the global nitrogen cycle: sources and consequences. Ecol. Appl..

[3] Galloway JN (2004). Nitrogen cycles: past, present and future. Biogeochemistry.

[4] Erisman JW, Sutton MA, Galloway J, Klimont Z, Winiwarter W (2008). How a century of ammonia synthesis changed the world. Nat. Geosci..

[5] Fowler D (2013). The global nitrogen cycle in the twenty-first century. Philos. Trans. R. Soc. Lond..

[6] Lin T (2014). Managing urban nutrient biogeochemistry for sustainable urbanization. Environ. Pollut..

[7] Groffman PM, Law NL, Belt KT, Band LE, Fisher GT (2004). Nitrogen fluxes and retention in urban watershed ecosystems. Ecosystems.

[8] Kaushal SS (2008). Interaction between urbanization and climate variability amplifies watershed nitrate export in Maryland. Environ. Sci. Technol..

[9] Erisman JW (2013). Consequences of human modification of the global nitrogen cycle. Philos. Trans. R. Soc. L. B Biol. Sci..

[10] Galloway JN (2008). Transformation of the nitrogen cycle: recent trends, questions, and potential solutions. Science.

[11] Galloway JN (2003). The nitrogen cascade. Bioscience.

[12] Schlesinger WH (2009). On the fate of anthropogenic nitrogen. Proc. Natl. Acad. Sci. USA.

[13] Fujimaki R, Sakai A, Kaneko N (2009). Ecological risks in anthropogenic disturbance of nitrogen cycles in natural terrestrial ecosystems. Ecol. Res..

[14] Gruber N, Galloway JN (2008). An Earth-system perspective of the global nitrogen cycle. Nature.

[15] Cui S, Shi Y, Groffman PM, Schlesinger WH, Zhu Y-G (2013). Centennial-scale analysis of the creation and fate of reactive nitrogen in China (1910-2010). Proc. Natl. Acad. Sci. USA.

[16] Dong Y, Xu L (2019). Aggregate risk of reactive nitrogen under anthropogenic disturbance in the Pearl River Delta urban agglomeration. J. Clean. Prod..

[17] Han Y, Li X, Nan Z (2011). Net anthropogenic nitrogen accumulation in the Beijing metropolitan region. Ecosystems.

[18] Kaye JP, Groffman PM, Grimm NB, Baker LA, Pouyat RV (2006). A distinct urban biogeochemistry?. Trends Ecol. Evol..

[19] Alberti M (2011). Research on coupled human and natural systems (CHANS): approach, challenges, and strategies. Bull. Ecol. Soc. Am..

[20] Færge J, Magid J, Penning De Vries FWT (2001). Urban nutrient balance for Bangkok. Ecol. Modell..

[21] Barles S (2007). Feeding the city: Food consumption and flow of nitrogen, Paris, 1801-1914. Sci. Total Environ..

[22] Jennifer F (2008). Nitrogen balance for the urban food metabolism of Toronto, Canada. Resour. Conserv. Recycl..

[23] Zhang Y, Lu H, Fath BD, Zheng H (2016). Modelling urban nitrogen metabolic processes based on ecological network analysis: A case of study in Beijing, China. Ecol. Modell..

[24] Gu B, Ju X, Chang J, Ge Y, Vitousek PM (2015). Integrated reactive nitrogen budgets and future trends in China. Proc. Natl. Acad. Sci. USA.

[25] Jiang S (2018). Enhanced nitrogen and phosphorus flows in a mixed land use basin: drivers and consequences. J. Clean. Prod..

[26] Shi Y, Cui S, Ju X, Cai Z, Zhu YG (2015). Impacts of reactive nitrogen on climate change in China. Sci. Rep..

[27] Guangzhou Municipal Statistics Bureau. *Guangzhou Statistical Yearbook* (National Bureau of Statistics of China Press, 2016).

[28] Environmental Status Bulletin of Guangzhou, Available at: www.gzepb.gov.cn/ (2016).

[29] Luo Z, Hu S, Chen D, Zhu B (2018). From production to consumption: a coupled human-environmental nitrogen flow analysis in China. Environ. Sci. Technol..

[30] Billen G (2016). Nitrogen use in the global food system: past trends and future trajectories of agronomic performance, pollution, trade, and dietary demand. Environ. Res. Lett..

[31] Ma L (2010). Modeling nutrient flows in the food chain of China. J. Environ. Qual..

[32] Doering, O. et al. Reactive Nitrogen in the United States: an analysis of inputs, flows, consequences, and management options. *United States Environ. Prot. Agency* (2011).

[33] de Vries, W., Leip, A. & Winiwarter, W. *Geographical variation in terrestrial nitrogen budgets across Europe* (Cambridge University Press, 2011).

[34] Miller G (2012). Getting minds out of the sewer. Science.

[35] Zhu X, Burger M, Doane TA, Horwath WR (2013). Ammonia oxidation pathways and nitrifier denitrification are significant sources of N_2_O and NO under low oxygen availability. Proc. Natl. Acad. Sci. USA.

[36] China Air Pollution Prevention and Control Action Plan. Available at: http://www.gov.cn (2013).

[37] Devol AH (2015). Denitrification, anammox, and N_2_ production in marine sediments. Ann. Rev. Mar. Sci..

[38] Li H, Mu H, Ming Z (2011). Analysis of China’s energy consumption impact factors. Procedia Environ. Sci..

[39] Xin, H. et al. A high-resolution ammonia emission inventory in China. *Global Biogeochem. Cycles* **26** (2012).

[40] Davidson EA, Keller M, Erickson HE, Verchot LV, Veldkamp E (2000). Testing a conceptual model of soil emissions of nitrous and nitric oxides. Bioscience.

[41] Mosier A (1998). Closing the global N_2_O budget: nitrous oxide emissions through the agricultural nitrogen cycle. Nutr. Cycl. Agroecosystems.

[42] Domene LAF, Ayres RU (2001). Nitrogen’s role in industrial systems. J. Ind. Ecol..

[43] Delucchi M (2000). Environmental externalities of motor-vehicle use in the US. J. Transp. Econ. Policy.

[44] Beevers SD (2012). Trends in NOx and NO_2_ emissions from road traffic in Great Britain. Atmos. Environ..

[45] Leach AM (2012). A nitrogen footprint model to help consumers understand their role in nitrogen losses to the environment. Environ. Dev..

[46] Zhijun H (2014). Study on degradation patterns of nitrogen in degrading dyes in a plug flow simulation reactor by TiO_2_. Environ. Enginnering.

[47] Gu B (2013). The role of industrial nitrogen in the global nitrogen biogeochemical cycle. Sci. Rep..

[48] Qing D, Keat S, Gersberg RM (2010). Municipal solid waste management in China: Status, problems and challenges. J. Environ. Manag..

[49] Li, H. *Synthesis of Azo dyes by Non-diazo-coupling Method* (Tianjin University of Technology, 2010).

[50] Xue X, Landis AE (2010). Eutrophication potential of food consumption patterns. Environ. Sci. Technol..

[51] Shibata H (2017). Nitrogen footprints: regional realities and options to reduce nitrogen loss to the environment. Ambio.

[52] Canfield DE, Glazer AN, Falkowski PG (2010). The evolution and future of Earth’s nitrogen cycle. Science.

[53] Liu X (2016). Intensification of phosphorus cycling in China since the 1600s. Proc. Natl. Acad. Sci. USA.

[54] Laner D, Rechberger H, Astrup T (2015). Systematic evaluation of uncertainty in material flow analysis. J. Ind. Ecol..

