# Supplementary material for: Aggravation of reactive nitrogen flow driven by human production and consumption in Guangzhou City China
Source: Nat Commun. 2020 Mar 5;11:1209. doi: 10.1038/s41467-020-14699-x (PMC7058066; doi:10.1038/s41467-020-14699-x)
Supplement: Supplementary file 1 — Supplementary Information [file 41467_2020_14699_MOESM1_ESM.pdf]

Supplementary Information to the article

Aggravation of Reactive Nitrogen Flow Driven by Human Production and Consumption in Guangzhou City, China

by Yue Dong et al.

Supplementary Figures

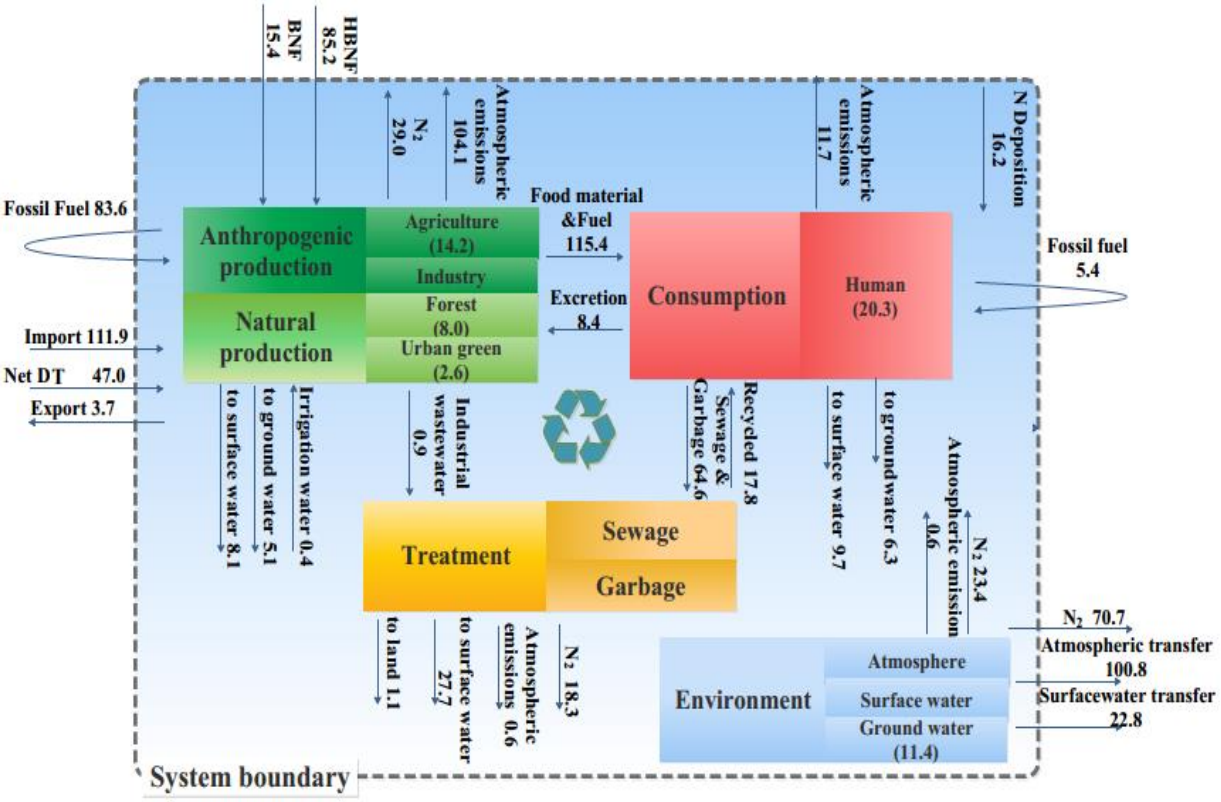

(a)

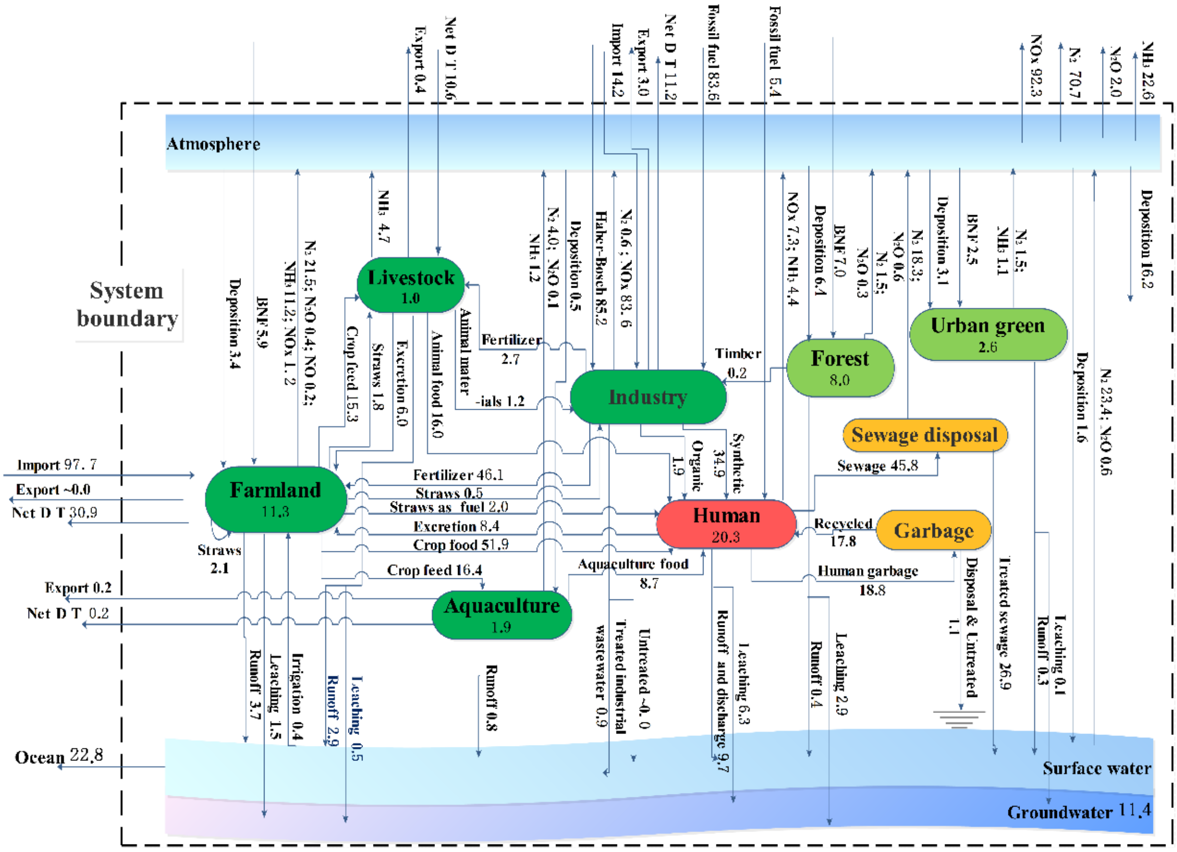

(b)

Supplementary Figure 1. (a) Coupled human-natural urban nitrogen flow analysis among four process groups (including 12 subsystems) in Guangzhou in 2015. (b) Detailed FNFA model framework and N fluxes among 12 subsystems within Guangzhou in 2015. The four process groups including production, consumption, treatment and environment are distinguished using the colors green, red, yellow, and blue, respectively. The numbers in brackets in each box represent Nr accumulation in each subsystem. The black dashed line represents the system boundary. BNF, biological N fixation; HBNF, Haber-Bosch N fixation; Net D T, net domestic trade. Units are in Gg N y^{-1}.

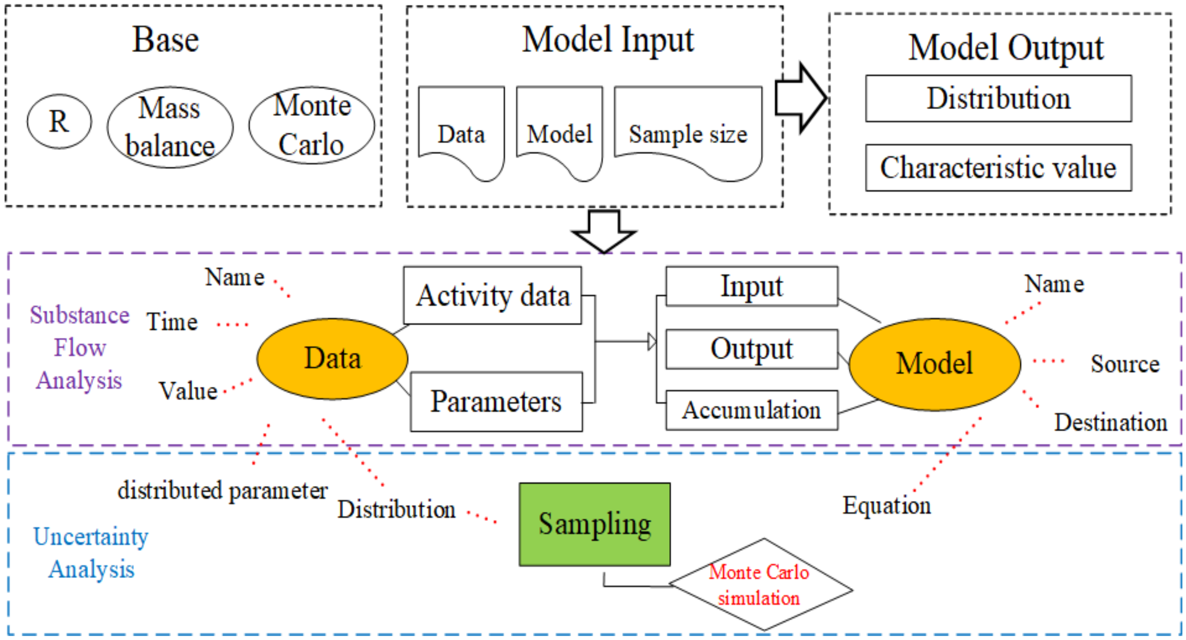

Supplementary Figure 2. Schematic diagram of the NNC.

.

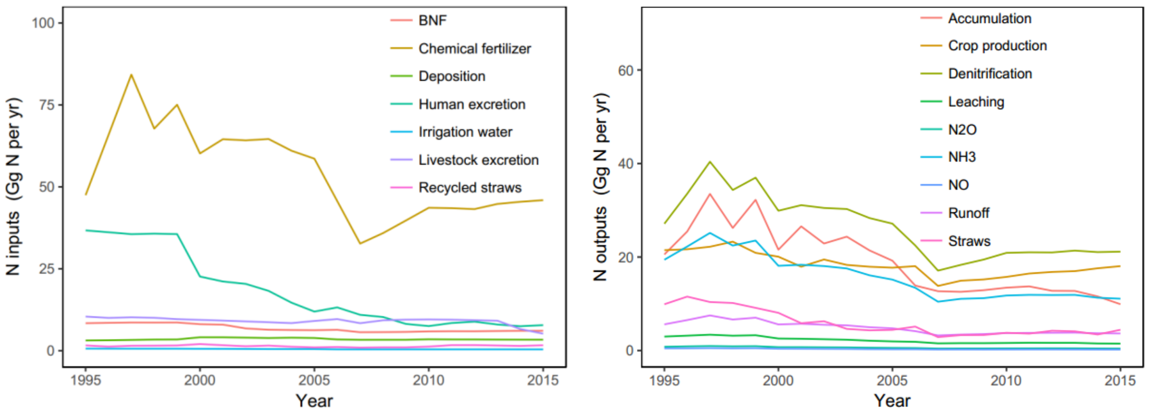

Supplementary Figure 3. N balance in farmland subsystem in Guangzhou from 1995 to 2015.

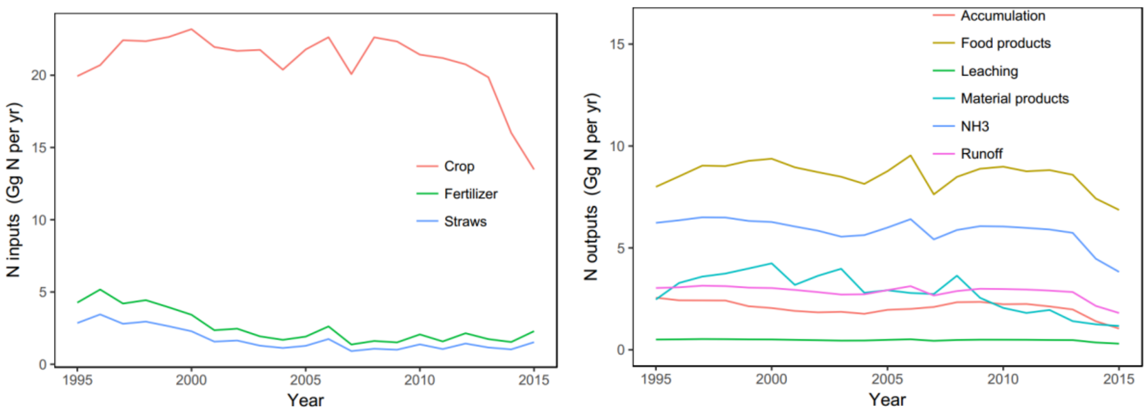

Supplementary Figure 4. N balance in livestock subsystem in Guangzhou from 1995 to 2015.

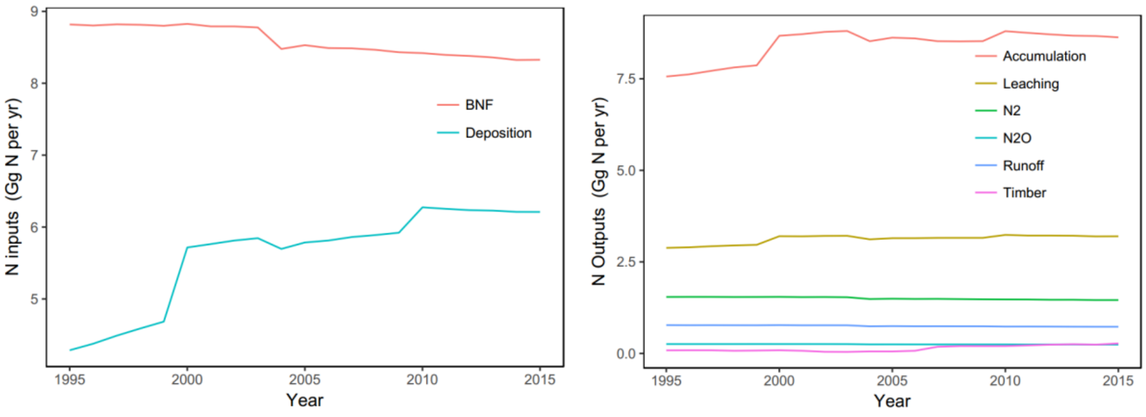

Supplementary Figure 5. N balance in forest subsystem in Guangzhou from 1995 to 2015.

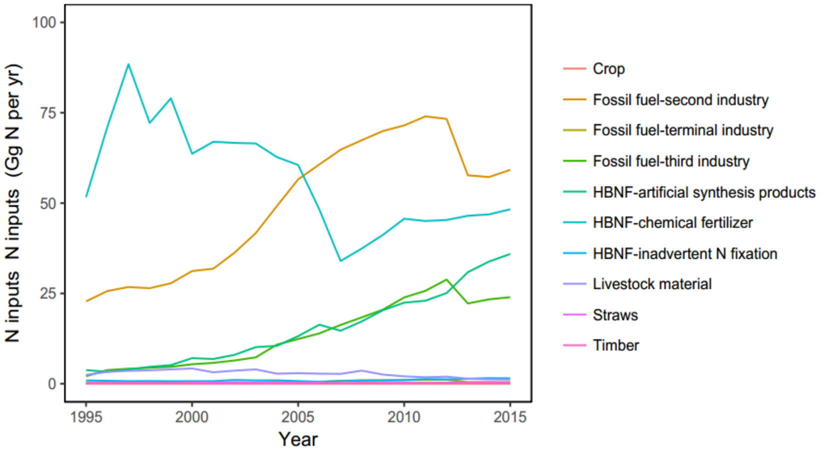

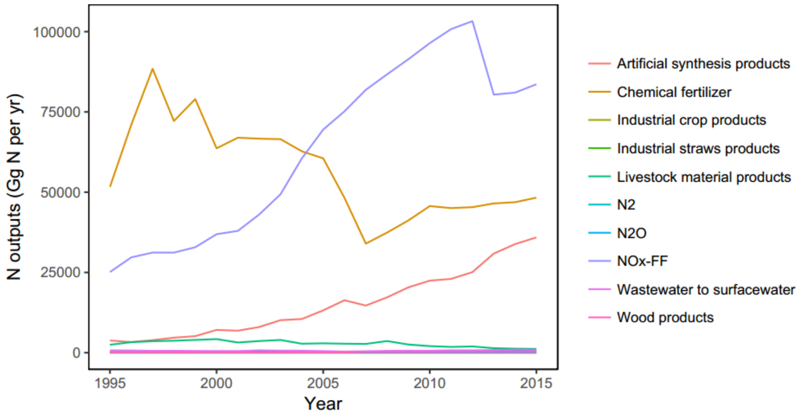

Supplementary Figure 6. N balance in industry subsystem in Guangzhou from 1995 to 2015.

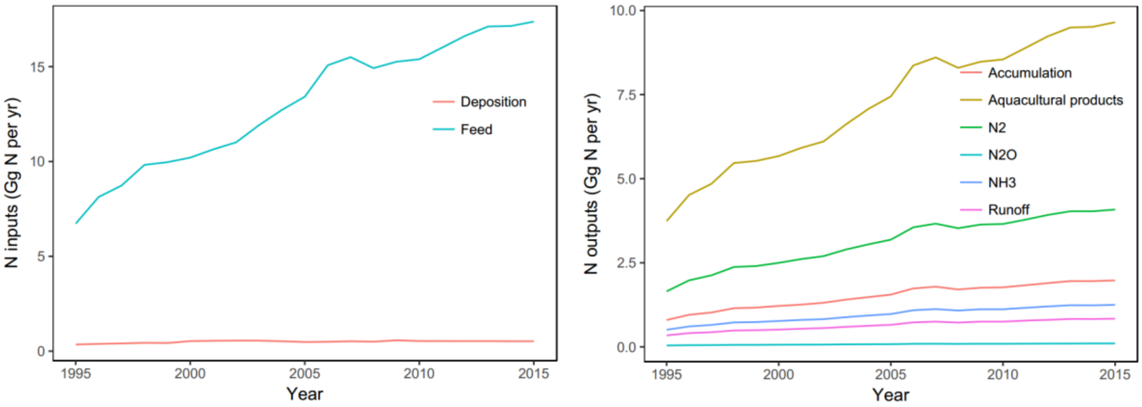

Supplementary Figure 7. N balance in aquaculture subsystem in Guangzhou from 1995 to 2015.

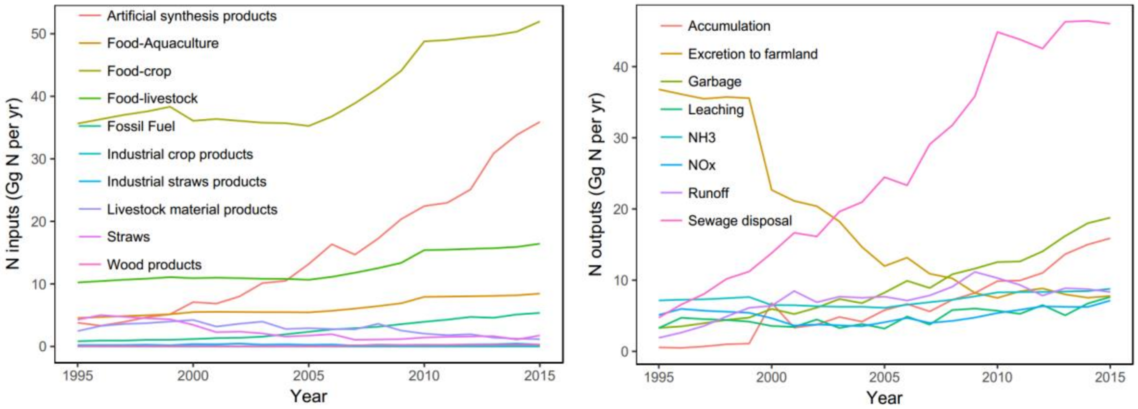

Supplementary Figure 8. N balance in human subsystem in Guangzhou from 1995 to 2015.

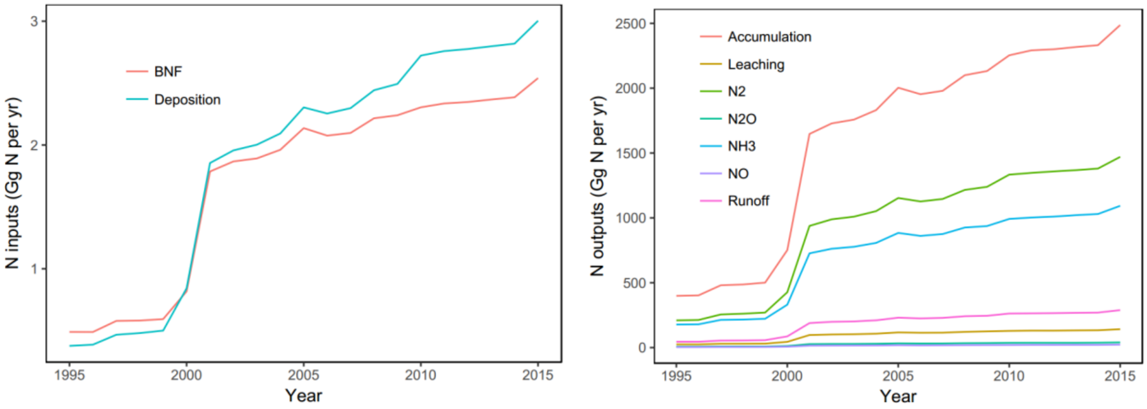

Supplementary Figure 9. N balance in urban green subsystem in Guangzhou from 1995 to 2015.

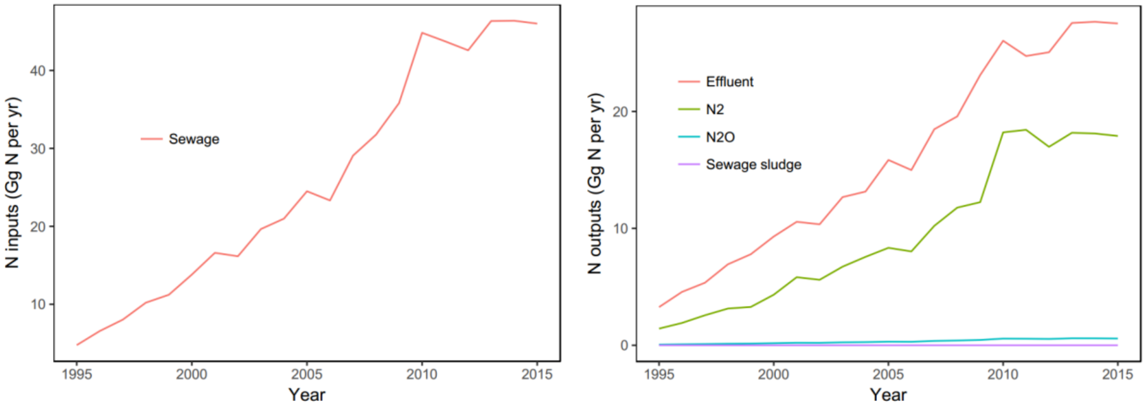

Supplementary Figure 10. N balance in sewage disposal subsystem in Guangzhou from 1995 to 2015.

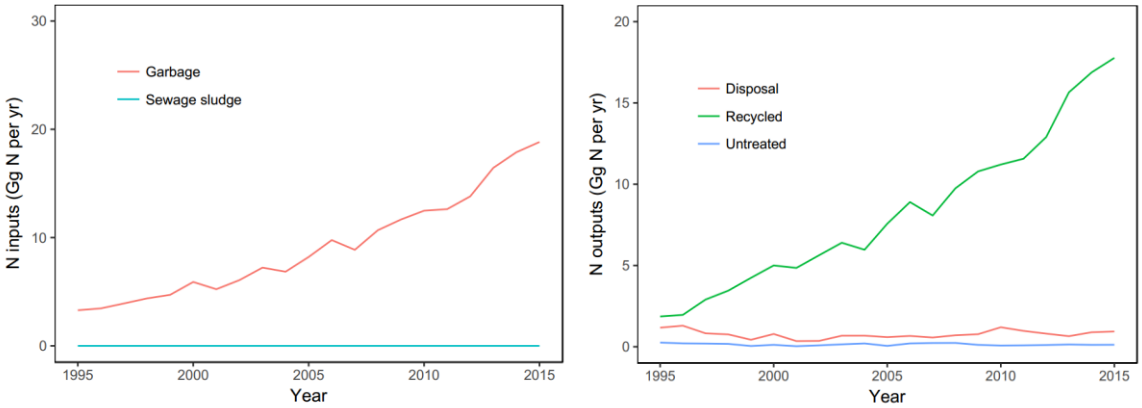

Supplementary Figure 11. N balance in garbage disposal in Guangzhou from 1995 to 2015.

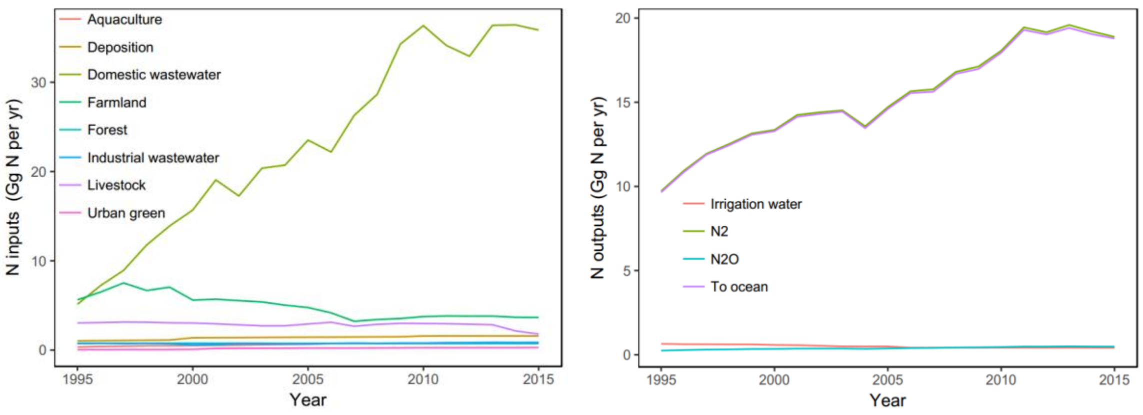

Supplementary Figure 12. N balance in surface water subsystem in Guangzhou from 1995 to 2015.

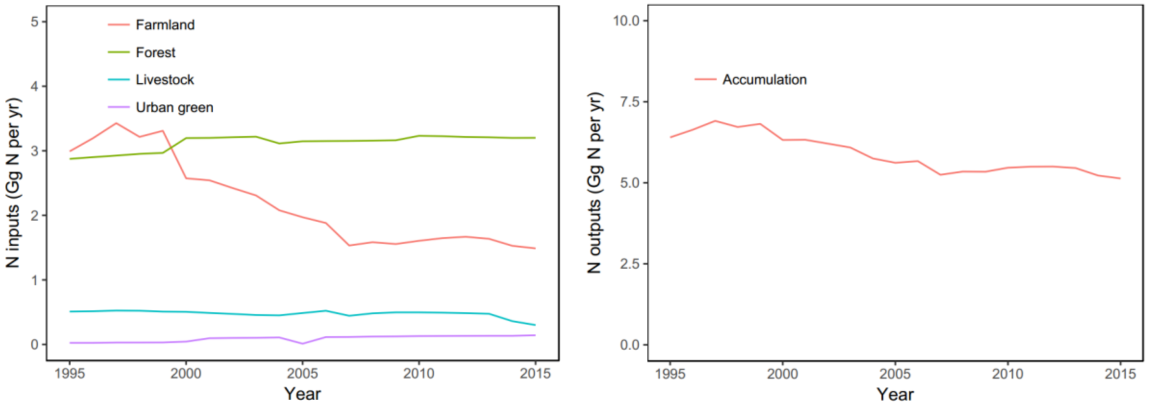

Supplementary Figure 13. N balance in groundwater subsystem in Guangzhou from 1995 to 2015.

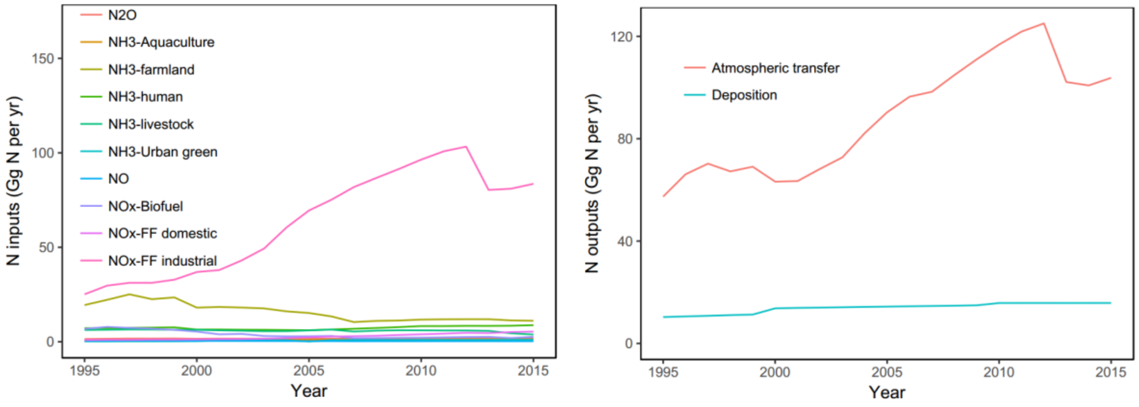

Supplementary Figure 14. N balance in atmosphere subsystem in Guangzhou from 1995 to 2015.

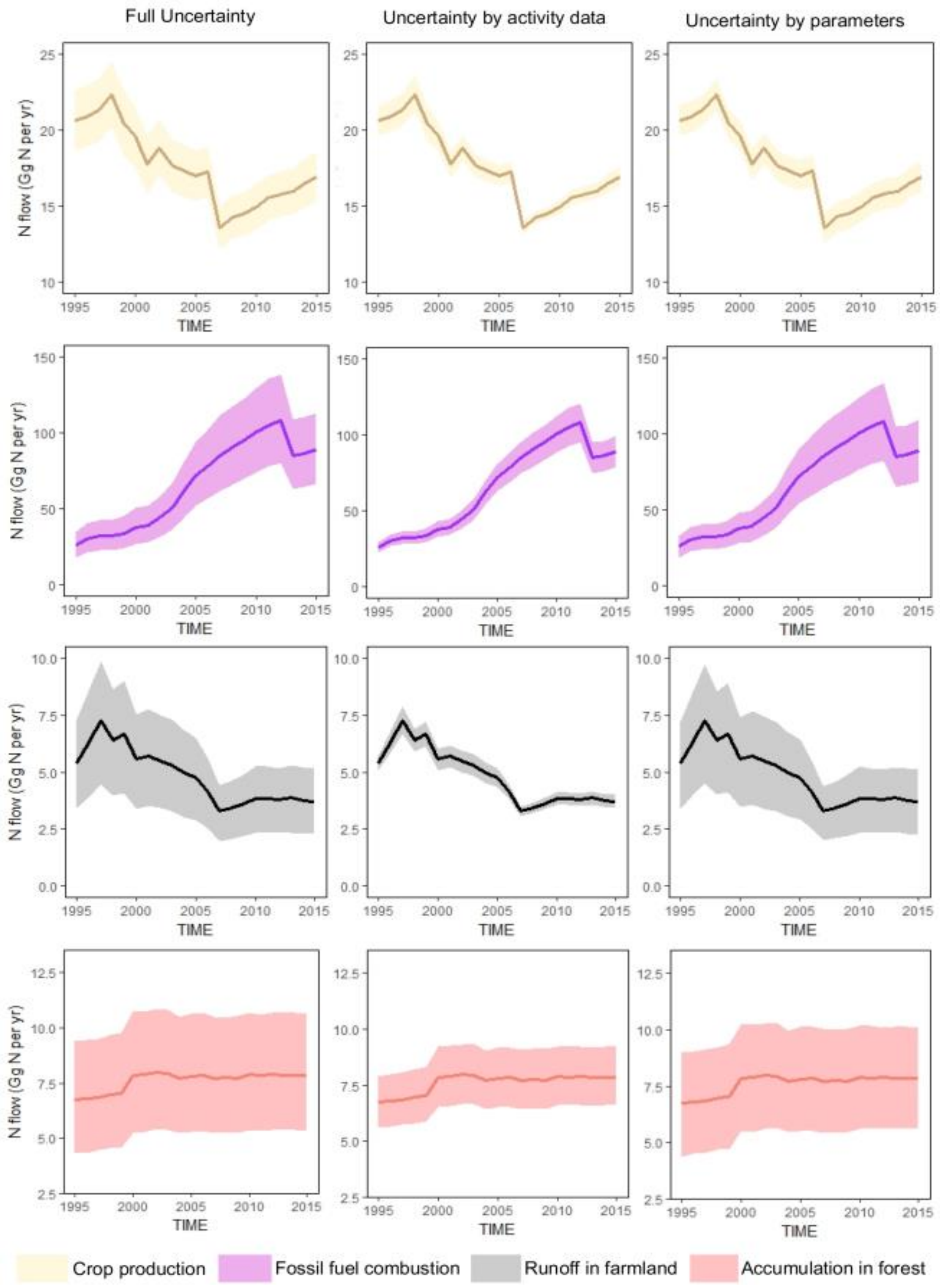

Supplementary Figure 15. Uncertainty of partial N flow using Monte Carlo simulation.

The annual N flows and uncertainties are shown as calculation value (lines) combined with

ranges between the 5th and 95th percentiles (shade areas). The left column presents the full uncertainties due to all activity data and parameters; the center column presents the uncertainties due to activity data while the right column presents the uncertainties due to parameters.

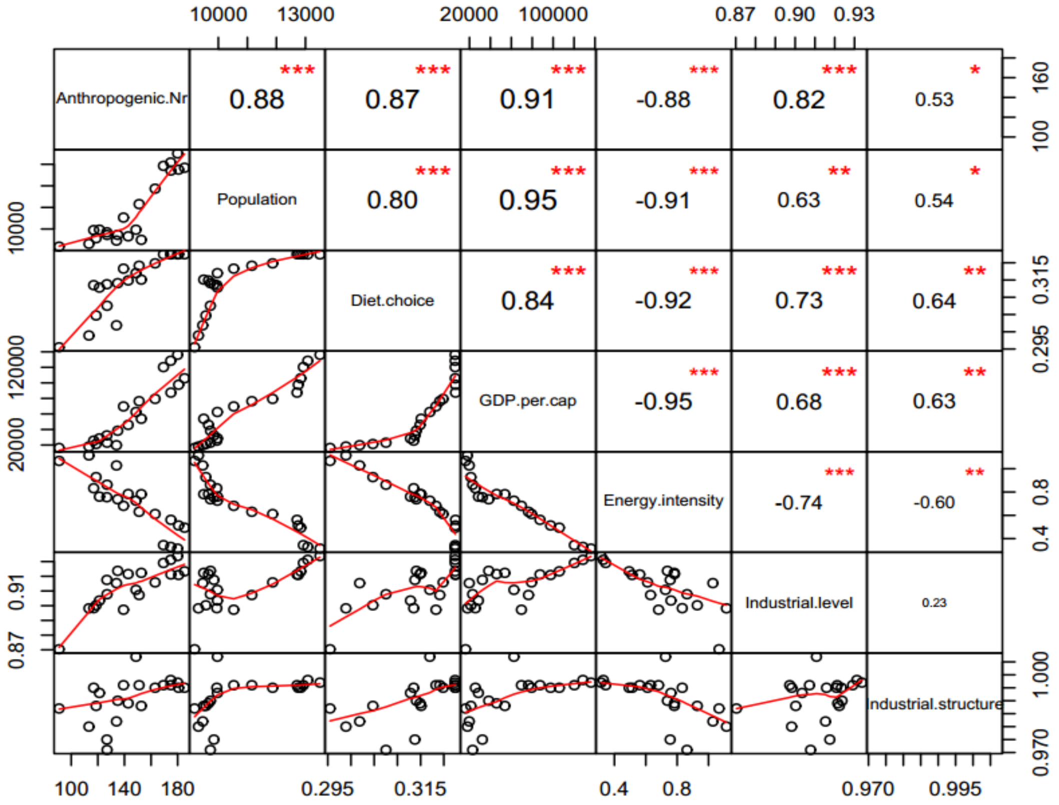

(a)

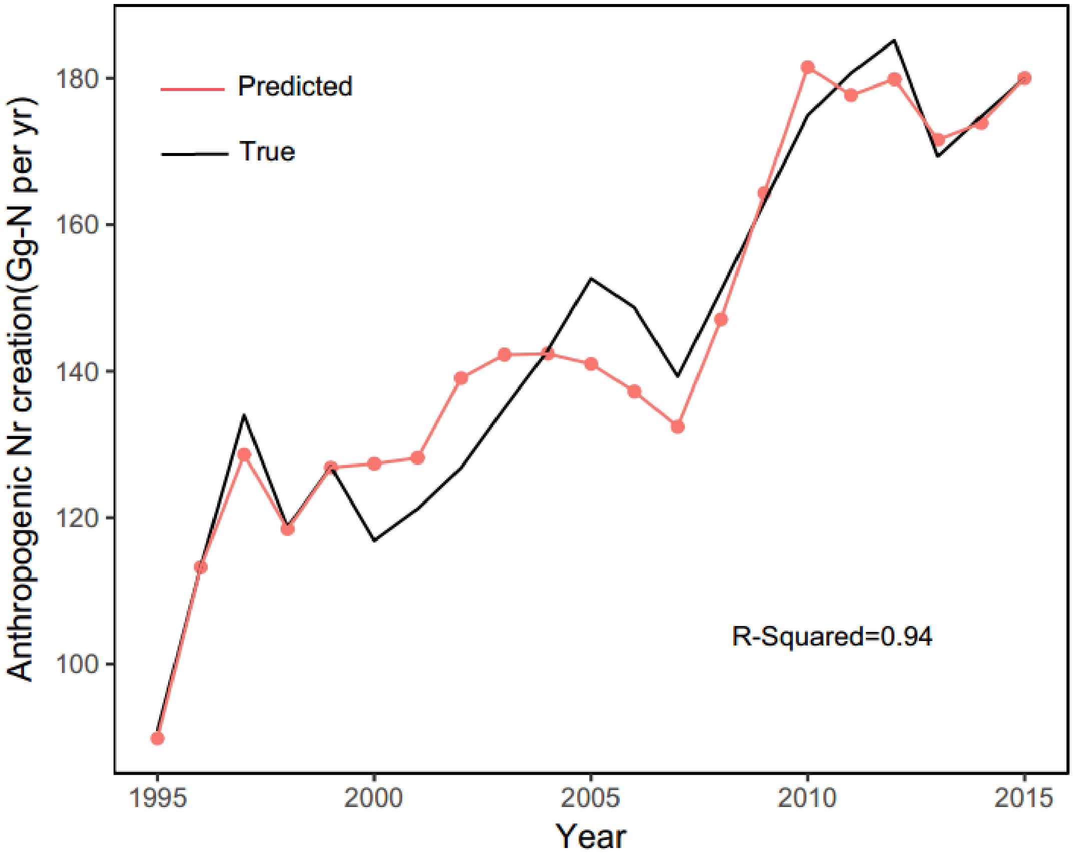

(b)

Supplementary Figure 16. Predicted anthropogenic Nr creation based on selected socioeconomic factors. (a) Correlation matrices of anthropogenic Nr creation and socioeconomic factors. Note: Pearson's correlation method was utilized to determine the correlation matrix. Each significance level is associated with a symbol: p-values (0.001, 0.01, 0.05, 0.1, and 1) and corresponding symbols ("***", "**", "*", ".", and " "). (b) Temporal trends of true and predicted anthropogenic Nr creation in Guangzhou.

Supplementary Tables

Supplementary Table 1. Mathematical equations of N balance in farmland subsystem.

Mathematical equations in Farmland	Activity data and parameters descriptions
$I_{dp}^{FL} = D_{fa}^{FL} \times P_{dp}^{R}$	D_{fa}^{FL} Farmland area<br>P_{dp}^{R} Rate of deposition
$I_{BNF}^{FL} = D_{sa-le}^{FL} \times P_{BNF-le}^{R} + D_{sa-pn}^{FL} \times P_{BNF-pn}^{R} + \left(D_{sa-t}^{FL} - D_{sa-le}^{FL} - D_{sa-pn}^{FL}\right) \times P_{BNF-ns}^{R}$	D_{sa-le}^{FL} Sown area of legume crops-legume<br>D_{sa-pn}^{FL} Sown area of legume crops-peanut<br>D_{sa-t}^{FL} Total sown area<br>P_{BNF-le}^{R} Symbiotic N fixation rate-legumes<br>P_{BNF-pn}^{R} Symbiotic N fixation rate-peanuts<br>P_{BNF-ns}^{R} Non-symbiotic N fixation rate
$I_{cf}^{FL} = D_{nf}^{ID} + D_{cf}^{ID} \times P_{cf}^{CT}$	D_{nf}^{ID} Consumption of chemical nitrogen fertilizer<br>D_{cf}^{ID} Consumption of chemical compound fertilizer<br>P_{cf}^{CT} N content of chemical compound fertilizer
$I_{he}^{FL} = D_{up}^{HM} \times P_{he}^{R} \times P_{uhe-fl}^{RO} + D_{rp}^{HM} \times P_{he}^{R} \times P_{rhe-fl}^{RO}$	D_{up}^{HM} Urban population

	D_{rp}^{HM} Rural population<br>P_{he}^{R} N excretion rate for human<br>P_{uhe-fl}^{RO} Ratio of human excretion returned to farmland-urban<br>P_{rhe-fl}^{RO} Ratio of human excretion returned to farmland-rural
$I_{le}^{FL} = \sum_i \left(D_{i-oh}^{LS} + D_{i-cl}^{LS} \right) \times P_{i-le}^{R} \times P_{i-fp}^{CS} \times P_{i-le-fl}^{RO} / 365$	D_{oh}^{LS} Number of livestock-on hand<br>D_{cl}^{LS} Number of livestock-column<br>P_{le}^{R} N excretion rate of livestock<br>P_{fp}^{CS} Feeding period of livestock<br>P_{le-fl}^{RO} Ratio of livestock excretion returned to farmland
$I_{ir}^{FL} = D_{ira}^{FL} \times P_{ir}^{CT}$	D_{ira}^{FL} Irrigation area<br>P_{ir}^{CT} N content of irrigation water
$I_{st}^{FL} = \sum_{i=1}^{7} D_{i-yc}^{FL} \times \left(1 / P_{i\text{-}hx}^{IX} - 1\right) \times P_{st}^{CT} \times P_{st-fl}^{RO}$	D_{yc}^{FL} Yield of crops<br>P_{hx}^{IX} Harvest index<br>P_{st}^{CT} N content of straws<br>P_{st-fl}^{RO} Straws recycled to farmland
$O_{cp}^{FL} = \sum_j D_{j-yc}^{FL} \times P_{j-cp}^{CT}$	D_{yc}^{FL} Yield of crops<br>P_{cp}^{CT} N content of crops
$O_{st}^{FL} = \sum_i D_{i-yc}^{FL} \times \left(1 / P_{i\text{-}hx}^{IX} - 1\right) \times P_{st}^{CT}$	D_{yc}^{FL} Yield of crops<br>P_{hx}^{IX} Harvest index<br>P_{st}^{CT} N content of straws
$O_{run}^{FL} = \left(I_{dp}^{FL} + I_{cf}^{FL} + I_{ir}^{FL} \right) \times P_{rusn-fl}^{R} + \left(I_{BNF}^{FL} + I_{he}^{FL} + I_{le}^{FL} + I_{st}^{FL} \right) \times P_{ruog-fl}^{R}$	$P_{rusn-fl}^{R}$ Runoff rate in farmland-synthetic N<br>$P_{ruog-fl}^{R}$ Runoff rate in farmland-

	organic N
$O_{lea}^{FL}=\left(I_{dp}^{FL}+I_{cf}^{FL}+I_{ir}^{FL}\right)\times P_{lesn-fl}^{R}+\left(I_{BNF}^{FL}+I_{he}^{FL}+I_{le}^{FL}+I_{st}^{FL}\right)\times P_{leog-fl}^{R}$	$P_{lesn-fl}^{R}$ Leaching loss rate in farmland- synthetic N<br>$P_{leog-fl}^{R}$ Leaching loss rate in farmland- organic N
$O_{N_2}^{FL}=\left(I_{dp}^{FL}+I_{cf}^{FL}+I_{ir}^{FL}\right)\times P_{N_2sn-fl}^{R}+\left(I_{BNF}^{FL}+I_{he}^{FL}+I_{le}^{FL}+I_{st}^{FL}\right)\times P_{N_2og-fl}^{R}$	$P_{N_2sn-fl}^{R}$ Denitrification(N_2) rate in farmland- synthetic N<br>$P_{N_2og-fl}^{R}$ Denitrification(N_2) rate in farmland- organic N
$O_{N_2O}^{FL}=\left(I_{dp}^{FL}+I_{cf}^{FL}+I_{ir}^{FL}\right)\times P_{N_2Osn-fl}^{R}+\left(I_{BNF}^{FL}+I_{he}^{FL}+I_{le}^{FL}+I_{st}^{FL}\right)\times P_{N_2Oog-fl}^{R}$	$P_{N_2Osn-fl}^{R}$ Denitrification(N_2O) rate in farmland- synthetic N<br>$P_{N_2Oog-fl}^{R}$ Denitrification(N_2O) rate in farmland- organic N
$O_{NO}^{FL}=\left(I_{dp}^{FL}+I_{cf}^{FL}+I_{ir}^{FL}\right)\times P_{NOsn-fl}^{R}+\left(I_{BNF}^{FL}+I_{he}^{FL}+I_{le}^{FL}+I_{st}^{FL}\right)\times P_{NOog-fl}^{R}$	$P_{NOsn-fl}^{R}$ NO emission rate in farmland- synthetic N<br>$P_{NOog-fl}^{R}$ NO emission rate in farmland- organic N
$O_{NH_3}^{FL}=\left(I_{dp}^{FL}+I_{cf}^{FL}\right)\times P_{NH_3sn-fl}^{R}+\left(I_{he}^{FL}+I_{le}^{FL}\right)\times P_{NH_3og-fl}^{R}$	$P_{NH_3sn-fl}^{R}$ NH_3 emission rate in farmland- synthetic N<br>$P_{NH_3og-fl}^{R}$ NH_3 emission rate in farmland- organic N
$O_{NO_x}^{FL}=\sum_i D_{i-yc}^{FL}\times\left(1/P_{i\text{-}hx}^{IX}-1\right)\times P_{st}^{CT}\times P_{st-bur}^{RO}$	D_{yc}^{FL} Yield of crops<br>P_{hx}^{IX} Harvest index<br>P_{st}^{CT} N content of straws<br>P_{st-bur}^{RO} Straws on-site combustion ratio

Supplementary Table 2. Mathematical equations of N balance in livestock subsystem

Mathematical equations in livestock	Activity data and parameter descriptions
$I_{st}^{LS} = \sum_i D_{i-yc}^{FL} \times \left(1/P_{i\text{-}hx}^{IX} - 1\right) \times P_{st}^{CT} \times P_{st-ls}^{RO}$	D_{yc}^{FL} Yield of crops<br>P_{hx}^{IX} Harvest index<br>P_{st}^{CT} N content of straws<br>P_{st-ls}^{RO} Straws to livestock feed
$I_{cp}^{LS} = \sum O^{LS} + AC^{LS} - I_{st}^{LS} - I_{cf}^{LS}$	See this table
$I_{cf}^{LS} = I_{st}^{LS} \times P_{ua}^{CS}$	P_{ua}^{CS} Urea input for straw ammoniation to produce food
$O_{lf}^{LS} = \sum_j D_{j-lf}^{LS} \times P_{j-lf}^{CT}$	D_{lf}^{LS} Livestock food production<br>P_{lf}^{CT} N content of livestock food
$O_{lm}^{LS} = \sum_j D_{j-lm}^{LS} \times P_{j-lm}^{CT}$	D_{lm}^{LS} Livestock material production<br>P_{lm}^{CT} N content of livestock material products
$O_{NH_3}^{LS} = \sum_j \left(D_{j-oh}^{LS} + D_{j-cl}^{LS}\right) \times P_{j-le}^{R} \times P_{j-fp}^{CS} \times P_{le-NH_3}^{RO} / 365$	D_{oh}^{LS} Number of livestock-on hand
$O_{run}^{LS} = \sum_j \left(D_{j-oh}^{LS} + D_{j-cl}^{LS}\right) \times \left(P_{j-le}^{R} - P_{tNH_3}^{RO}\right) \times P_{j-fp}^{CS} \times P_{le-run}^{RO} / 365$	D_{cl}^{LS} Number of livestock-column
$O_{lea}^{LS} = \sum_j \left(D_{j-oh}^{LS} + D_{j-cl}^{LS}\right) \times \left(P_{j-le}^{R} - P_{tNH_3}^{RO}\right) \times P_{j-fp}^{CS} \times P_{le-lea}^{RO} / 365$	P_{le}^{R} N excretion rate of livestock<br>P_{fp}^{CS} Feeding period of livestock<br>$P_{le-NH_3}^{RO}$ NH_3 volatilization rate of livestock excretion

	$P_{tNH_3}^{RO}$ Total NH_3 of livestock excretion
	P_{le-run}^{RO} Runoff rate of livestock excretion
	P_{le-lea}^{RO} Leaching rate of livestock excretion
$AC^{LS}=\sum_{k=1}^{5}D_{k-oh}^{LS}\times P_{k-bw}^{CS}\times P_{k-np}^{CT}\times P_{k-lf}^{CT}$	D_{oh}^{LS} Number of livestock-on hand
	P_{bw}^{CS} Body weight of livestock
	P_{np}^{CT} Neat percentage
	P_{lf}^{CT} N content of livestock food

Supplementary Table 3. Mathematical equations of N balance in forest subsystem

Mathematical equations in forest	Activity data and parameter descriptions
$I_{dp}^{FR}=D_{fa}^{FR}\times P_{dp}^{R}$	D_{fa}^{FR} Forest area
	P_{dp}^{R} Rate of deposition
$I_{BNF}^{FR}=D_{fa}^{FR}\times P_{BNF-fr}^{R}$	P_{BNF-fr}^{R} N fixation rate of forest
$O_{ft}^{FR}=D_{wp}^{FR}\times P_{wo}^{CT}$	D_{wp}^{FR} Wood production
	P_{wo}^{CT} N content of wood
$O_{run}^{FR}=D_{fa}^{FR}\times P_{run-fr}^{R}$	P_{run-fr}^{R} Runoff rate of forest
$O_{N_2O}^{FR}=D_{fa}^{FR}\times P_{N_2O-fr}^{R}$	$P_{N_2O-fr}^{R}$ N_2O emission rate of forest
$O_{N_2}^{FR}=D_{fa}^{FR}\times P_{N_2-fr}^{R}$	$P_{N_2-fr}^{R}$ Denitrification(N_2) rate of forest
$O_{lea}^{FR}=\left(I_{dp}^{FR}+I_{BNF}^{FR}\right)\times P_{lea-fr}^{R}$	P_{lea-fr}^{R} Leaching rate of forest

Supplementary Table 4. Mathematical equations of N balance in industry subsystem

**Mathematical equations in industry**	**Activity data and parameter descriptions**
$I_{HBNF}^{ID} = I_{cf}^{FL} + I_{cf}^{LS} + \sum_j D_{j-asp}^{ID} \times P_{j-asp}^{CT} + D_{wwnl}^{ID}$	D_{asp}^{ID} Artificial synthesis production<br>D_{wwnl}^{ID} NH_3-N number of industrial wastewater discharge<br>P_{asp}^{CT} N content of artificial synthesis products
$I_{st}^{ID} = \sum_i D_{i-yc}^{FL} \times \left(1/P_{i\text{-}hx}^{IX} - 1\right) P_{st}^{CT} \times P_{st-im}^{RO}$	D_{yc}^{FL} Yield of crops<br>P_{hx}^{IX} Harvest index<br>P_{st}^{CT} N content of straws<br>P_{st-im}^{RO} Straws as industrial material
$I_{cp}^{ID} = \sum_i D_{i-yc}^{FL} \times P_{i-cp}^{CT}$	D_{yc}^{FL} Yield of crops<br>P_{cp}^{CT} N content of crops
$I_{lm}^{ID} = O_{lm}^{LS}$	See Supplementary Table 2
$I_{ft}^{ID} = O_{ft}^{FR}$	See Supplementary Table 3
$I_{ff}^{ID} = \sum D_{ff}^{ID} \times P_{ff}^{R}$	D_{ff}^{ID} Consumption of fossil fuels<br>P_{ff}^{R} NOx emission factor of fossil fuel
$O_{fer}^{ID} = I_{cf}^{FL} + I_{cf}^{LS}$	See Supplementary Table 1-2
$O_{idp-og}^{ID} = I_{lm}^{ID} + I_{ft}^{ID} + I_{st}^{ID} + I_{cp}^{ID}$	See this table

$O_{idp-asp}^{ID} = \sum_j D_{j-asp}^{ID} \times P_{j-asp}^{CT}$	D_{asp}^{ID} Artificial synthesis production<br>P_{asp}^{CT} N content of artificial synthesis products
$O_{ww}^{ID} = D_{wwnl}^{ID} \times \left(1 - D_{wdr}^{ID}\right) + D_{wwnl}^{ID} \times D_{wdr}^{ID} \times \left(1 - P_{deN_2}^{R} - P_{deN_2O}^{R}\right)$	D_{wwnl}^{ID} NH_3-N number of industrial wastewater discharge<br>D_{wdr}^{ID} Disposal rate of industrial wastewater<br>$P_{deN_2}^{R}$ Denitrification rate(N_2) of sewage<br>$P_{deN_2O}^{R}$ Denitrification rate(N_2O) of sewage
$O_{NOx}^{ID} = I_{ff}^{ID}$	
$O_{N_2}^{ID} = D_{wwnl}^{ID} \times D_{wdr}^{ID} \times P_{deN_2}^{R}$	
$O_{N_2O}^{ID} = D_{wwnl}^{ID} \times D_{wdr}^{ID} \times P_{deN_2O}^{R}$	

Supplementary Table 5. Mathematical equations of N balance in aquaculture subsystem

Mathematical equations in aquaculture	Activity data and parameter descriptions
$I_{dp}^{AQ} = D_{aa}^{AQ} \times P_{dp}^{R}$	D_{aa}^{AQ} Aquaculture area<br>P_{dp}^{R} Rate of deposition
$I_{cp}^{AQ} = D_{ap}^{AQ} \times P_{ap}^{CT} \times P_{apf}^{RO}$	D_{ap}^{AQ} Aquaculture production
$O_{ap}^{AQ} = D_{ap}^{AQ} \times P_{ap}^{CT}$	P_{ap}^{CT} N content of aquaculture products<br>P_{apf}^{RO} Ratio of feed to aquaculture products
$O_{N_2}^{AQ} = \left(I_{dp}^{AQ} + I_{cp}^{AQ} - O_{ap}^{AQ}\right) \times P_{N_2-aq}^{R}$	$P_{N_2-aq}^{R}$ Denitrification rate of aquaculture
$O_{N_2O}^{AQ} = \left(I_{dp}^{AQ} + I_{cp}^{AQ} - O_{ap}^{AQ}\right) \times P_{N_2O-aq}^{R}$	$P_{N_2O-aq}^{R}$ N_2O emission rate of aquaculture
$O_{NH_3}^{AQ} = \left(I_{dp}^{AQ} + I_{cp}^{AQ} - O_{ap}^{AQ}\right) \times P_{NH_3-aq}^{R}$	$P_{NH_3-aq}^{R}$ NH_3 rate of aquaculture
$O_{run}^{AQ} = \left(I_{dp}^{AQ} + I_{cp}^{AQ} - O_{ap}^{AQ}\right) \times P_{run-aq}^{R}$	P_{run-aq}^{R} Runoff rate of aquaculture

Supplementary Table 6. Mathematical equations of N balance in human subsystem

Mathematical equations in human	Activity data and parameter descriptions
$I_{cp}^{HM}=D_{up}^{HM}\times P_{ucc}^{R}+D_{rp}^{HM}\times P_{rcc}^{R}$	D_{up}^{HM} Urban population
$I_{lf}^{HM}=D_{up}^{HM}\times P_{ulc}^{R}+D_{rp}^{HM}\times P_{rlc}^{R}$	D_{rp}^{HM} Rural population
$I_{ap}^{HM}=D_{up}^{HM}\times P_{uac}^{R}+D_{rp}^{HM}\times P_{rac}^{R}$	P_{ucc}^{R} Crops consumption rate-urban residents<br>P_{rcc}^{R} Crops consumption rate-rural residents<br>P_{ulc}^{R} Livestock food consumption rate-urban residents<br>P_{rlc}^{R} Livestock food consumption rate-rural residents<br>P_{uac}^{R} Aquaculture products consumption rate-urban residents<br>P_{rac}^{R} Aquaculture products consumption rate-rural residents
$I_{idp-og}^{HM}=O_{idp-og}^{ID}$	See Supplementary Table 4
$I_{idp-asp}^{HM}=O_{idp-asp}^{ID}$	See Supplementary Table 4
$I_{ff}^{HM}=\sum D_{ff}^{HM}\times P_{ff}^{R}$	D_{ff}^{HM} Consumption of fossil fuels<br>P_{ff}^{R} NOx emission factor of fossil fuel
$I_{stbu}^{HM}=\sum_{i} D_{i-yc}^{FL}\times\left(1/P_{i\text{-}hx}^{IX}-1\right)\times P_{st}^{CT}\times P_{st-de}^{RO}$	D_{yc}^{FL} Yield of crops<br>P_{hx}^{IX} Harvest index<br>P_{st}^{CT} N content of straws<br>P_{st-de}^{RO} Straws as domestic energy
$O_{tds}^{HM}=D_{dsnl}^{HM}\times D_{dwdr}^{SD}$	D_{dsnl}^{HM} NH_3-N number of domestic sewage discharge

$O_{utds}^{HM} = D_{dsnl}^{HM} \times \left(1 - D_{dwdr}^{SD}\right)$	D_{dwdr}^{SD} Disposal rate of domestic wastewater
$O_{he}^{HM} = I_{he}^{FL}$	See Supplementary Table 1
$O_{NO_x}^{HM} = I_{ff}^{HM} + I_{stbu}^{HM}$	See this table
$O_{NH_3}^{HM} = D_{up}^{HM} \times P_{NH_3-uh}^{R} + D_{rp}^{HM} \times P_{NH_3-rh}^{R}$	D_{up}^{HM} Urban population<br>D_{rp}^{HM} Rural population<br>$P_{NH_3-uh}^{R}$ NH_3 emission factor-urban residents<br>$P_{NH_3-rh}^{R}$ NH_3 emission factor-rural residents
$O_{gb}^{HM} = \left(I_{idp-og}^{HM} + I_{idp-asp}^{HM}\right) \times P_{gba}^{R}$	P_{gba}^{R} Industrial products abandon rate

Supplementary Table 7. Mathematical equations of N balance in urban green subsystem

Mathematical equations in urban green	Activity data and parameter descriptions
$I_{dp}^{UG} = D_{ga}^{UG} \times P_{dp}^{R}$	D_{ga}^{UG} Urban green area
$I_{BNF-ug}^{UG} = D_{ga}^{UG} \times P_{BNF-ug}^{R}$	P_{dp}^{R} Rate of deposition
	P_{BNF-ug}^{R} N fixation rate of urban green
$O_{run}^{UG} = I_{dp}^{UG} \times P_{rusn-fl}^{R} + I_{BNF-ug}^{UG} \times P_{ruog-fl}^{R}$	$P_{rusn-fl}^{R}$ Runoff rate - synthetic N
	$P_{ruog-fl}^{R}$ Runoff rate - organic N
$O_{lea}^{UG} = I_{dp}^{UG} \times P_{lesn-fl}^{R} + I_{BNF-ug}^{UG} \times P_{leog-fl}^{R}$	$P_{lesn-fl}^{R}$ Leaching loss rate - synthetic N
	$P_{leog-fl}^{R}$ Leaching loss rate - organic N
$O_{N_2}^{UG} = I_{dp}^{UG} \times P_{N_2sn-fl}^{R} + I_{BNF-ug}^{UG} \times P_{N_2og-fl}^{R}$	$P_{N_2sn-fl}^{R}$ Denitrification(N_2) rate - synthetic N
	$P_{N_2og-fl}^{R}$ Denitrification(N_2) rate - organic N
$O_{N_2O}^{UG} = I_{dp}^{UG} \times P_{N_2Osn-fl}^{R} + I_{BNF-ug}^{UG} \times P_{N_2Oog-fl}^{R}$	$P_{N_2Osn-fl}^{R}$ Denitrification(N_2O) rate - synthetic N
	$P_{N_2Oog-fl}^{R}$ Denitrification(N_2O) rate - organic N
$O_{NO}^{UG} = I_{dp}^{UG} \times P_{NOsn-fl}^{R} + I_{BNF-ug}^{UG} \times P_{NOog-fl}^{R}$	$P_{NOsn-fl}^{R}$ NO emission rate - synthetic N
	$P_{NOog-fl}^{R}$ NO emission rate - organic N
$O_{NH_3}^{UG} = I_{dp}^{UG} \times P_{NH_3sn-fl}^{R} + I_{BNF-ug}^{UG} \times P_{NH_3og-fl}^{R}$	$P_{NH_3sn-fl}^{R}$ NH_3 emission rate - synthetic N
	$P_{NH_3og-fl}^{R}$ NH_3 emission rate - organic N

Supplementary Table 8. Mathematical equations of N balance in sewage disposal subsystem

Mathematical equations in sewage disposal	Activity data and parameter descriptions
$I_{ds}^{SD} = O_{tds}^{HM}$	See Supplementary Table 6
$O_{N_2}^{SD} = I_{ds}^{SD} \times P_{deN_2}^{R}$	$P_{deN_2}^{R}$ Denitrification rate(N_2) of sewage
$O_{N_2O}^{SD} = I_{ds}^{SD} \times P_{deN_2O}^{R}$	$P_{deN_2O}^{R}$ Denitrification rate(N_2O) of sewage
$O_{ss}^{SD} = I_{ds}^{SD} \times P_{slu}^{R} \times P_{slu}^{CT} \times \left(1 - P_{moi}^{CT}\right)$	P_{slu}^{R} Generation rate of sludge<br>P_{slu}^{CT} N content of sludge<br>P_{moi}^{CT} Moisture content of sludge
$O_{ste}^{SD} = I_{ds}^{SD} \times \left(1 - P_{deN_2}^{R} - P_{deN_2O}^{R}\right) - O_{ss}^{SD}$	$P_{deN_2}^{R}$ Denitrification rate(N_2) of sewage<br>$P_{deN_2O}^{R}$ Denitrification rate(N_2O) of sewage

Supplementary Table 9. Mathematical equations of N balance in garbage disposal subsystem

Mathematical equations in garbage disposal	Activity data and parameter descriptions
$I_{hg}^{GD} = O_{gb}^{HM}$	See Supplementary Table 6
$I_{ss}^{GD} = O_{ss}^{SD}$	See Supplementary Table 8
$O_{gut}^{GD} = \left(I_{hg}^{GD} + I_{ss}^{GD}\right) \times D_{ur}^{GB}$	D_{ur}^{GB} Utilization rate of garbage
$O_{gdl}^{GD} = \left(I_{hg}^{GD} + I_{ss}^{GD}\right) \times D_{dr}^{GB} \times P_{gblf}^{RO}$	D_{dr}^{GB} Disposal rate of garbage
$O_{gdc}^{GD} = \left(I_{hg}^{GD} + I_{ss}^{GD}\right) \times D_{dr}^{GB} \times P_{gbdi}^{RO}$	P_{gblf}^{RO} Landfill ratio of garbage<br>P_{gbdi}^{RO} Composting ratio of garbage
$O_{got}^{GD} = I_{hg}^{GD} + I_{ss}^{GD} - O_{gut}^{GD} - O_{gdl}^{GD} - O_{gdc}^{GD}$	See this table

Supplementary Table 10. Mathematical equations of N balance in surface water subsystem

Mathematical equations in surface water	Activity data and parameter descriptions
$I_{dp}^{SW} = D_{wa}^{SW} \times P_{dp}^{R}$	D_{wa}^{SW} Area of surface water<br>P_{dp}^{R} Rate of deposition
$O_{ir}^{SW} = I_{ir}^{FL}$	See Supplementary Table 1
$O_{N_2}^{SW} = IN^{SW} \times P_{N_2-sw}^{R}$	$P_{N_2-sw}^{R}$ Denitrification(N_2) rate in surface water
$O_{N_2O}^{SW} = IN^{SW} \times P_{N_2O-sw}^{R}$	$P_{N_2O-sw}^{R}$ Denitrification(N_2O) rate in surface water

Supplementary Table 11. Mathematical equations of N balance in atmosphere subsystem

Mathematical equations in atmosphere	Activity data and parameter description
$O_{dp}^{AT} = D_{ra} \times P_{dp}^{R}$	D_{ra} Area of Study area<br>P_{dp}^{R} Rate of deposition

Supplementary Table 12. Mathematical equations of systemic balance and crosscheck

Mathematical equations in balance and crosscheck	Activity data and parameter description
$IM^{cp} = \sum D_{IM}^{CP} \times P_{cp}^{CT}$	D_{IM}^{CP} Crop food import
$EX^{cp} = \sum D_{EX}^{CP} \times P_{cp}^{CT}$	D_{EX}^{CP} Crop food export
$IM^{lf} = \sum D_{IM}^{lf} \times P_{lf}^{CT}$	D_{IM}^{lf} livestock food import
$EX^{lf} = \sum D_{EX}^{lf} \times P_{lf}^{CT}$	D_{EX}^{lf} livestock food export
$IM^{ap} = \sum D_{IM}^{ap} \times P_{ap}^{CT}$	D_{IM}^{ap} Aquaculture food import
$EX^{ap} = \sum D_{EX}^{ap} \times P_{ap}^{CT}$	D_{EX}^{ap} Aquaculture food export<br>P_{cp}^{CT} N content of crops<br>P_{lf}^{CT} N content of livestock food<br>P_{ap}^{CT} N content of aquaculture products
$IM^{ip} = \sum D_{IM}^{ip} \times P_{asp}^{CT}$	D_{IM}^{ip} Industrial products import
$EX^{ip} = \sum D_{EX}^{ip} \times P_{ap}^{CT}$	D_{EX}^{ip} Industrial products export<br>P_{asp}^{CT} N content of artificial synthesis products

Supplementary Table 13 Description of activity data with uncertainty assumption

Activity data	Description	Unit	Distribution	Sources	CV
**Farmland**					
D_{fa}^{FL}	Farmland area	ha	Uniform	[1]	±0.1
D_{sa-le}^{FL}	Sown area of legume crops-legume	ha	Uniform	[1]	±0.1
D_{sa-pn}^{FL}	Sown area of legume crops-peanut	ha	Uniform	[1]	±0.1
D_{sa-t}^{FL}	Total sown area	ha	Uniform	[1]	±0.1
D_{ira}^{FL}	Irrigation area	ha	Uniform	[1]	±0.1
D_{yc}^{FL}	Yield of crops (Rice, potato, soybean, sugarcane, peanut, cotton, tobacco, vegetables, banana, citrus, pineapple, melons, litchi, longan, date, hemp, tea)	t	Uniform	[1,2]	±0.1
**Livestock**					
D_{oh}^{LS}	Number of livestock-on hand (Pig, cattle, sheep, poultry, rabbit, horse, birds, chicken, duck, goose)	cap	Uniform	[1,2]	±0.1
D_{cl}^{LS}	Number of livestock-column	cap	Uniform	[1,2]	±0.1
D_{lf}^{LS}	Livestock food production (Pork, beef, lamb, poultry, rabbit, egg, milk, honey, chicken, duck, goose, game meat)	t	Uniform	[1,2]	±0.1

D_{lm}^{LS}	Livestock material production (Leather, silk, wool, sheepskin, cowhide, pigskin)	t	Uniform	[1,2]	±0.1
**Forest**					
D_{fa}^{FR}	Forest area	ha	Uniform	[1]	±0.1
D_{wp}^{FR}	Wood production	t	Uniform	[1]	±0.1
**Industry**					
D_{nf}^{ID}	Consumption of chemical nitrogen fertilizer	t	Uniform	[1,2]	±0.1
D_{cf}^{ID}	Consumption of chemical compound fertilizer	t	Uniform	[1,2]	±0.1
D_{asp}^{ID}	Artificial synthesis production (Detergent, drugs, plastic, synthetic fiber, rubber, chemical pesticide, nitrate, synthetic dyes, industrial explosives)	t	Uniform	[1]	±0.1
D_{ff}^{ID}	Consumption of fossil fuels	t (SEC)	Uniform	[1]	±0.1
D_{wwnl}^{ID}	NH_3-N number of industrial wastewater discharge	t	Uniform	[3,4,5]	±0.2
D_{wdr}^{ID}	Disposal rate of industrial wastewater	%	Uniform	[1]	±0.2
**Aquaculture**					
D_{aa}^{AQ}	Aquaculture area	ha	Uniform	[1]	±0.1
D_{ap}^{AQ}	Aquaculture production	ha	Uniform	[1]	±0.1
**Human**					

D_{up}^{HM}	Urban population	thousand	Uniform	1	±0.1
D_{rp}^{HM}	Rural population	thousand	Uniform	1	±0.1
D_{ff}^{HM}	Consumption of fossil fuels	t (SEC)	Uniform	1	±0.1
D_{dsnl}^{HM}	NH_3-N number of domestic sewage discharge	t	Uniform	3,4,5	±0.2
**Urban green**					
D_{ga}^{UG}	Urban green area	ha	Uniform	1	±0.1
**Sewage disposal**					
D_{dwdr}^{SD}	Disposal rate of domestic wastewater	%	Uniform	1	±0.2
**Garbage disposal**					
D_{dr}^{GB}	Disposal rate of garbage	%	Uniform	1, 4	±0.2
D_{ur}^{GB}	Utilization rate of garbage	%	Uniform	1, 4	±0.2
**Surface water**					
D_{wa}^{SW}	Area of surface water	ha	Uniform	1	±0.1
**Study area**					
D_{ra}	Area of study area	ha	Uniform	1	±0.1

Supplementary Table 14 Description of parameters with uncertainty assumption

Parameters	Description	Unit	Value	Distribution	Sources	CV
**Farmland**						
P^{R}_{BNF-le}	Symbiotic N fixation rate-legumes	kg N ha^{-1} y^{-1}	105	Triangular (70,115,130)	[6,7]	
P^{R}_{BNF-pn}	Symbiotic N fixation rate-peanuts	kg N ha^{-1} y^{-1}	112	Triangular (72,124,140)	[6]	
P^{R}_{BNF-ns}	Non-symbiotic N fixation rate	kg N ha^{-1} y^{-1}	18.75	Triangular (15,16.25,25)	[7,8]	
P^{IX}_{hx}	Harvest index	-	Table 15	Normal	[9,10]	±0.2
P^{CT}_{st}	N content of straws	%	Table 15	Normal	[11]	±0.1
P^{RO}_{st-fl}	Straws recycled to farmland	%	Table 16	Uniform	[1,4,12,13,]	±0.3
P^{CT}_{ir}	N content of irrigation water	kg N ha^{-1} y^{-1}	5.2	Uniform (3.4,7.0)	[14]	±0.2
P^{CT}_{cp}	N content of crops	%	Table 15	Normal	[15]	±0.1
$P^{R}_{rusn-fl}$	Runoff rate in farmland- synthetic N	%	Table 17	Uniform	[16,17,18,19]	±0.3
$P^{R}_{ruog-fl}$	Runoff rate in farmland- organic N	%	Table 17	Uniform	[16,17,18,19]	±0.3

$P^{R}_{lesn-fl}$	Leaching loss rate in farmland- synthetic N	%	Table 17	Uniform	[16,17,18,19]	±0.3
$P^{R}_{leog-fl}$	Leaching loss rate in farmland- organic N	%	Table 17	Uniform	[16,17,18,19]	±0.3
$P^{R}_{N_2sn-fl}$	Denitrification(N_2) rate in farmland- synthetic N	%	Table 17	Uniform	[16,17,18,19]	±0.3
$P^{R}_{N_2og-fl}$	Denitrification(N_2) rate in farmland- organic N	%	Table 17	Uniform	[16,17,18,19]	±0.3
$P^{R}_{N_2Osn-fl}$	Denitrification(N_2O) rate in farmland- synthetic N	%	Table 17	Uniform	[16,17,18,19]	±0.3
$P^{R}_{N_2Oog-fl}$	Denitrification(N_2O) rate in farmland- organic N	%	Table 17	Uniform	[16,17,18,19]	±0.3
$P^{R}_{NOsn-fl}$	NO emission rate in farmland- synthetic N	%	Table 17	Uniform	[16,17,18,19]	±0.3
$P^{R}_{NOog-fl}$	NO emission rate in farmland- organic N	%	Table 17	Uniform	[16,17,18,19]	±0.3

$P^{R}_{NH_3sn-fl}$	NH_3 emission rate in farmland- synthetic N	%	Table 17	Uniform	[16,17,18,19]	±0.3
$P^{R}_{NH_3og-fl}$	NH_3 emission rate in farmland- organic N	%	Table 17	Uniform	[16,17,18,19]	±0.3
P^{RO}_{st-bur}	Straws on-site combustion ratio	%	Table 16	Uniform	[1,4,12,13]	±0.3
**Livestock**						
P^{RO}_{le-fl}	Ratio of livestock excretion returned to farmland	%	Pig: 60;<br>cattle: 77.7;<br>sheep: 70;<br>poultry: 39;<br>Rabbit:40	Uniform (39,81);<br>Uniform (51,100);<br>Uniform (46,94);<br>Uniform (25,53);<br>Uniform (26,54)	[14]	±0.2
P^{RO}_{st-ls}	Straws to livestock feed	%	Table 16	Uniform	[1,4,12,13]	±0.3
P^{CS}_{ua}	Urea input for straw ammoniation to produce food	--	1.5	Uniform (0.98,2.02)	[20]	±0.2
P^{CT}_{lf}	N content of livestock food	%	Table 19	Normal	[21,22]	±0.1
P^{CT}_{lm}	N content of livestock	%	Table 19	Normal	[21,22]	±0.1

	material products					
P_{le}^{R}	N excretion rate of livestock	kg cap^{-1} y^{-1}	Table 18	Triangular	[23,24,25]	
P_{fp}^{CS}	Feeding period of livestock	day	Table 18	Normal	[26,27]	±0.1
$P_{le-NH_3}^{RO}$	NH_3 volatilization rate of livestock excretion	%	33	Uniform (21.6,44.4)	[25,28]	±0.2
$P_{tNH_3}^{RO}$	Total NH_3 of livestock excretion	kg cap^{-1} y^{-1}	Table 18	Uniform	[24,25,28,29]	±0.2
P_{le-run}^{RO}	Runoff rate of livestock excretion	%	30	Uniform (14.4,45.6)	[24,30]	±0.3
P_{le-lea}^{RO}	Leaching rate of livestock excretion	%	5	Uniform (2.4,7.6)	[31]	±0.3
P_{bw}^{CS}	Body weight of livestock	kg	Table 18	Normal	[26]	±0.1
P_{np}^{CT}	Neat percentage	%	Table 18	Uniform	[26]	±0.1
**Forest**						
P_{BNF-fr}^{R}	N fixation rate of forest	kg N ha^{-1} y^{-1}	24	Triangular (12,26.9,33.1)	[32,33]	
P_{wo}^{CT}	N content of wood	%	0.2	Triangular(0.1,0.2,0.4)	[34,35]	

P_{run-fr}^{R}	Runoff rate of forest	kg N ha^{-1} y^{-1}	1.5	Triangular(0.71,1,2.79)	[32,36]	
$P_{N_2O-fr}^{R}$	N_2O emission rate of forest	kg N ha^{-1} y^{-1}	1	Triangular(0.7,1.1,1.2)	[36]	
$P_{N_2-fr}^{R}$	Denitrification(N_2) rate of forest	kg N ha^{-1} y^{-1}	5	Triangular(3.6,5,6.4)	[36]	
P_{lea-fr}^{R}	Leaching rate of forest	%	22	Uniform (10.6,33.4)	[37,38]	±0.3
**Industry**						
P_{cf}^{CT}	N content of chemical compound fertilizer	%	30	Uniform (19.6,40.4)	[15]	±0.2
P_{asp}^{CT}	N content of artificial synthesis products	%	Table 21	Normal	[31]	±0.2
P_{st-im}^{RO}	Straws as industrial material	%	Table 16	Uniform	[1,4,12,13]	±0.3
P_{ff}^{R}	NOx emission factor of fossil fuel	kg N t^{-1}	Table 20	Uniform	[39]	±0.2
P_{gba}^{R}	Industrial products abandon rate	%	50	Uniform(32.7,67.3)	[20]	±0.2
**Aquaculture**						

P_{ap}^{CT}	N content of aquaculture products	%	2.6<br>Table 19	Triangular (2.34,2.50,2.97)	[40,41,42,43]	
P_{apf}^{RO}	Ratio of feed to aquaculture products	--	1.8	Triangular(1.5,1.90,2.0)	[40,41,42,]	
$P_{N_2-aq}^{R}$	Denitrification rate of aquaculture	%	50	Uniform(24.0,75.9)	[31]	±0.3
$P_{N_2O-aq}^{R}$	N_2O emission rate of aquaculture	%	1.25	Uniform(0.60,1.90)	[18]	±0.3
$P_{NH_3-aq}^{R}$	NH_3 rate of aquaculture	%	15	Uniform(7.2,22.8)	[44]	±0.3
P_{run-aq}^{R}	Runoff rate of aquaculture	%	10	Uniform(4.80,15.2)	[45]	±0.3
**Human**						
P_{ucc}^{R}	Crops consumption rate-urban residents	kg cap^{-1} y^{-1}	95-00s:3.5<br>00-10s:3.8<br>10-15s:3.9	Triangular(3.4, 3.5, 3.6)<br>Triangular(3.7,3.8,3.9)<br>Triangular(3.8,3.9,4.0)	[1,4,46]	±0.2
P_{rcc}^{R}	Crops consumption rate-rural residents	kg cap^{-1} y^{-1}	95-00s:3.2<br>00-10s:3.4<br>10-15s:3.5	Triangular(3.1,3.2,3.3)<br>Triangular(3.3,3.4,3.5)<br>Triangular(3.4,3.5,3.6)	[1,4,46]	±0.2

P_{ulc}^{R}	Livestock food consumption rate-urban residents	kg cap^{-1} y^{-1}	95-00s:1.04<br>00-10s:1.14<br>10-15s:1.20	Triangular (0.98, 1.04, 1.1)<br>Triangular (1.08, 1.14, 1.2)<br>Triangular (1.15, 1.19, 1.26)	[1,4,46]	±0.2
P_{rlc}^{R}	Livestock food consumption rate-rural residents	kg cap^{-1} y^{-1}	95-00s:0.95<br>00-10s:1.05<br>10-15s:1.10	Triangular (0.9, 0.95, 1)<br>Triangular (0.99, 1.05, 1.11)<br>Triangular (1.08, 1.10, 1.12)	[1,4,46]	±0.2
P_{uac}^{R}	Aquaculture products consumption rate-urban residents	kg cap^{-1} y^{-1}	95-00s:0.50<br>00-10s:0.55<br>10-15s:0.65	Triangular (0.43, 0.50, 0.59),<br>Triangular (0.45, 0.55, 0.65)<br>Triangular (0.6, 0.65, 0.7)	[1,4,46]	±0.2

P_{rac}^{R}	Aquaculture products consumption rate-rural residents	kg cap^{-1} y^{-1}	95-00s:0.45<br>00-10s:0.50<br>10-15s:0.60	Triangular (0.38, 0.44, 0.5)<br>Triangular (0.44, 0.5, 0.56)<br>Triangular (0.55, 0.60, 0.65)	[1,4,46]	±0.2
P_{st-de}^{RO}	Straws as domestic energy	%	Table 16	Uniform	[1,4,12,13]	±0.3
$P_{NH_3-uh}^{R}$	NH_3 emission factor-urban residents	kg cap^{-1} y^{-1}	0.3	Uniform(0.20,0.40)	[47]	±0.2
$P_{NH_3-rh}^{R}$	NH_3 emission factor-rural residents	kg cap^{-1} y^{-1}	0.5	Uniform(0.33,0.67)	[47]	±0.2
P_{he}^{R}	N excretion rate for human	kg cap^{-1} y^{-1}	95-00s:4.5<br>00-10s:5.0<br>10-15s:5.2	Triangular (4.2,4.5, 4.8)<br>Triangular(4.8, 5.0, 5.2)<br>Triangular (4.9,5.2,5.5)	[23]	±0.2
P_{uhe-fl}^{RO}	Ratio of human excretion returned to farmland-urban	%	95-00s:60<br>00-10s:20<br>10-15s:5	Uniform (38.6,79.4)<br>Uniform (13.2,27.2)<br>Uniform (3.27,6.73)	[5,48]	±0.2
P_{rhe-fl}^{RO}	Ratio of human excretion returned to	%	95-00s:80<br>00-10s:60	Uniform (51.7,100.0)<br>Uniform (39.3,80.9)	[5,48]	±0.2

	farmland-rural		10-15s:50	Uniform (32.7,67.3)		
**Urban green**						
P^{R}_{BNF-ug}	N fixation rate of urban green	kg N ha^{-1} y^{-1}	18	Uniform (11.8,24.23)	[49]	±0.2
**Sewage disposal**						
$P^{R}_{deN_2}$	Denitrification rate(N_2) of sewage	%	95-00s:30<br>00-10s:35<br>10-15s:40	Uniform (19.6,40.4)<br>Uniform (22.9,47.1)<br>Uniform (26.1,53.9)	[50,51]	±0.2
$P^{R}_{deN_2O}$	Denitrification rate(N_2O) of sewage	%	1.25	Uniform(0.82,1.68)	[18]	±0.2
P^{R}_{slu}	Generation rate of sludge	%	0.035	Triangular (0.020,0.042,0.043)	[52,53]	
P^{CT}_{slu}	N content of sludge	%	2.9	Triangular (1.9,2.0,4.8)	[27,54,55]	
P^{CT}_{moi}	Moisture content of sludge	%	80	Triangular (70,72,98)	[53]	
**Garbage disposal**						
P^{RO}_{gblf}	Landfill ratio of garbage	%	80	Uniform (60,100)	[55,56,57,58]	±0.3
P^{RO}_{gbdi}	Composting ratio of garbage	%	20	Uniform (9.6,30.4)	[55,56,57,58]	±0.3

**Surface water**						
$P_{N_2-sw}^{R}$	Denitrification(N_2) rate in surface water	%	50	Uniform(24.0,75.9)	[51]	±0.3
$P_{N_2O-sw}^{R}$	Denitrification(N_2O) rate in surface water	%	1.25	Uniform(0.60,1.90)	[18]	±0.3
**Atmosphere**						
P_{dp}^{R}	Rate of deposition	kg N ha^{-1} y^{-1}	95-00s:12.7<br>00-10s:18.6<br>10-15s:21.8	Triangular (10.9,11.2,16.0)<br>Triangular (15.3,19.1,21.3)<br>Triangular (20.5,22.0,22.9)	[59,60,61,62]	±0.2

Supplementary Table 15 N content of crops and straws and harvest index

Type	N content of crops P_{cp}^{FL} (%)	N content of straws P_{st}^{CT} (%)	Harvest index P_{hx}^{IX}
Rice	1.12<br>Normal (1.12,0.013)	0.91<br>Normal (0.91,0.008)	0.53<br>Normal (0.53,0.011)
Potato	1.04<br>Normal (1.04,0.011)	2.51<br>Normal (2.51,0.063)	0.66<br>Normal (0.66,0.017)
Soybean	3.15<br>Normal (3.15,0.099)	2.10<br>Normal (2.10,0.044)	0.50<br>Normal (0.50,0.010)
Sugarcane	0.37<br>Normal (0.37,0.001)	1.10<br>Normal (1.10,0.012)	0.67<br>Normal (0.67,0.018)
Peanut	3.25<br>Normal (3.25,0.106)	1.82<br>Normal (1.82,0.033)	0.55<br>Normal (0.55,0.012)
Cotton	1.01<br>Normal (1.01,0.010)	1.24<br>Normal (1.24,0.015)	0.25<br>Normal (0.25,0.002)
Tobacco	1.42<br>Normal (1.42,0.020)	1.44<br>Normal (1.44,0.021)	0.50<br>Normal (0.50,0.010)
Hemp	0.8<br>Normal (0.8,0.006)	0.8<br>Normal (0.8,0.006)	0.36<br>Normal (0.36, 0.005)
Vegetables	0.24<br>Normal (0.24,0.001)	--	--

Banana	0.22<br>Normal (0.22,0.0005)	--	--
Cirtus	0.11<br>Normal (0.11,0.0001)	--	--
Pineapple	0.22<br>Normal (0.22,0.0005)	--	--
Melons	0.16<br>Normal (0.16,0.0003)	--	--
Litchi	0.20<br>Normal (0.20,0.0004)	--	--
Longan	0.20<br>Normal (0.20,0.0004)	--	--
Date	0.43<br>Normal (0.43,0.002)		
Tea	3.5<br>Normal (3.5,0.123)		

Supplementary Table 16 Fate of straws in different periods

**Fare of straws(%)**	**1995-2000**	**2000-2010**	**2010-2015**
Recycled to farmland P_{st-fl}^{RO}	13<br>Uniform(6.2,19.8)	22<br>Uniform(10.6,33.4)	28<br>Uniform(13.5,42.5)
Livestock feed P_{st-ls}^{RO}	25<br>Uniform(12.0,38.0)	23<br>Uniform(11.0,35.0)	24<br>Uniform(11.5,36.5)
Industrial material P_{st-im}^{RO}	2<br>Uniform(1.0,3.0)	5<br>Uniform(2.4,7.6)	6<br>Uniform(2.9,9.1)
Domestic energy P_{st-de}^{RO}	39<br>Uniform(18.7,59.3)	31<br>Uniform(14.9,47.1)	26<br>Uniform(12.5,39.5)
On-site combustion P_{st-bur}^{RO}	21<br>Uniform(10.1,31.9)	19<br>Uniform(9.1,28.9)	16<br>Uniform(7.7,24.3)

Supplementary Table 17 Nitrogen loss rate in farmland

**Nitrogen loss rate (%)**	**Synthetic N**	**Organic N**
Runoff ($P^R_{rusn-fl}$, $P^R_{ruog-fl}$)	5.2<br>Uniform (2.50,7.90)	5.0<br>Uniform (2.40,7.60)
Leaching ($P^R_{lesn-fl}$, $P^R_{leog-fl}$)	1.2<br>Uniform (0.58,1.82)	4.0<br>Uniform (1.92,6.08)
Denitrification(N_2) ($P^R_{N_2sn-fl}$, $P^R_{N_2og-fl}$)	36.4<br>Uniform (17.49,55.31)	15.0<br>Uniform (7.21,22.79)
Denitrification(N_2O) ($P^R_{N_2Osn-fl}$, $P^R_{N_2Oog-fl}$)	0.4<br>Uniform (0.19,0.61)	1.0<br>Uniform (0.48,1.52)
NO emission ($P^R_{NOsn-fl}$, $P^R_{NOog-fl}$)	0.1<br>Uniform (0.05,0.15)	0.7<br>Uniform (0.33,1.06)
NH_3 volatilization ($P^R_{NH_3sn-fl}$, $P^R_{NH_3og-f}$)	16.0<br>Uniform (7.69,24.31)	23.0<br>Uniform (11.0,35.0)

Supplementary Table 18 Livestock N parameters

Type	Body weight (kg) P_{bw}^{CS}	Neat percentage (%) P_{np}^{CT}	N excretion rate (kg N cap^{-1} y^{-1}) P_{le}^{R}	Feeding period (day) P_{fp}^{CS}	Total NH_3 of excretion(kg cap^{-1}y^{-1}) $P_{tNH_3}^{RO}$
Pig	100<br>Uniform<br>(83,117)	0.7<br>Uniform<br>(0.58,0.82)	8<br>Triangular(4.87,8,11.23)	199<br>Uniform(165,233)	2.33<br>Uniform(1.52,3.14)
Cattle	500<br>Uniform<br>(413,587)	0.45<br>Uniform<br>(0.37,0.53)	40<br>Triangular(34.13,40,45.87)	365<br>Uniform(302,428)	18.6<br>Uniform(12.2,25.0)
Sheep	80<br>Uniform<br>(66,94)	0.5<br>Uniform<br>(0.41,0.59)	11.23<br>Triangular(5,11.23,17.46)	365<br>Uniform(302,428)	4.18<br>Uniform(2.73,5.63)
Poultry	2<br>Uniform<br>(1.7,2.3)	0.8<br>Uniform<br>(0.66,0.94)	0.3<br>Triangular(0.1,0.33,0.47)	55<br>Uniform(45,65)	0.1<br>Uniform(0.07,0.13)
Rabbit	2.5<br>Uniform<br>(2.1,2.9)	0.5<br>Uniform<br>(0.41,0.59)	0.45<br>Triangular(0.4,0.45,0.5)	120<br>Uniform(99,141)	0.2<br>Uniform(0.13,0.27)
Horse	500	0.5	68.6439	365	18.6

	Uniform<br>(413,587)	Uniform<br>(0.41,0.59)	Triangular(52,68.64,85.28)	Uniform(302,428)	Uniform(12.2,25.0)
Chicken	1.5<br>Uniform<br>(1.24,1.76)	0.8<br>Uniform<br>(0.66,0.94)	0.09<br>Uniform(0.74,1.05)	45<br>Uniform(37,53)	0.04<br>Uniform(0.03,0.06)
Duck	2.5<br>Uniform<br>(2.07,2.93)	0.8<br>Uniform<br>(0.66,0.94)	0.47<br>Uniform(0.39,0.55)	50<br>Uniform(41,59)	0.25<br>Uniform(0.16,0.34)
Goose	3<br>Uniform<br>(2.48,3.52)	0.8<br>Uniform<br>(0.66,0.94)	0.47<br>Uniform(0.39,0.55)	70<br>Uniform(58,82)	0.25<br>Uniform(0.16,0.34)

Supplementary Table 19 N content of livestock and aquaculture products

**Food products**	**N content (%)** P_{lf}^{CT}	**Material products**	**N content (%)** P_{lm}^{CT}
Pork	2.1 Normal (2.1,0.04)	Leather	12.6 Normal (12.6,1.59)
Beef	3.2 Normal (3.2,0.10)	Silk	3.4 Normal (3.4,0.12)
Lamb	2.0 Normal (2.0,0.04)	Wool	12.2 Normal (12.2,1.49)
Poultry	3.0 Normal (3.0,0.10)	Sheepskin	8.4 Normal (8.4， 0.71)
Rabbit	3.2 Normal (3.2,0.10)	Cowhide	12.6 Normal (12.6,1.59)
Eggs	2.1 Normal (2.1,0.04)	Pigskin	10.1 Normal (10.1,1.02)
Milk	0.5 Normal (0.5,0.002)		
Honey	0.1 Normal (0.1,0.0001)		
Chicken	3.1 Normal (3.1,0.10)		
Duck	2.5 Normal (2.5,0.06)		
Goose	2.9 Normal (2.9, 0.08)		
Game meat	2.9 Normal (2.9, 0.08)		
Fish	3.0 Normal (3.0, 0.09)		
Shrimp and crab	2.9 Normal (2.9, 0.08)		
Shellfish	2.1 Normal (2.1, 0.04)		
Algae products	3.7 Normal (3.7, 0.14)		
Other aquaculture products	2.9 Normal (2.9, 0.08)		

Supplementary Table 20 NOx emission factors by sources and fuel types (kg t^{-1})

P_{ff}^{R}	**Coal**	**Coke**	**Crude oil**	**Gasoline**	**Kerosene**	**Diesel**	**Natural gas**[a]
Industry	2.3<br>Uniform<br>(1.5,3.1)	2.7<br>Uniform<br>(1.8,3.6)	1.5<br>Uniform<br>(1.0,2.0)	5.1<br>Uniform<br>(3.3,6.9)	2.3<br>Uniform<br>(1.5,3.1)	2.9<br>Uniform<br>(1.9,3.9)	0.6<br>Uniform<br>(0.4,0.8)
Commerce	1.1<br>Uniform<br>(0.7,1.5)	1.4<br>Uniform<br>(0.9,1.9)	0.9<br>Uniform<br>(0.6,1.1)	5.1<br>Uniform<br>(3.3,6.9)	1.4<br>Uniform<br>(0.9,1.9)	0.8<br>Uniform<br>(0.5,1.1)	0.4<br>Uniform<br>(0.3,0.5)
Construction	2.3<br>Uniform<br>(1.5,3.1)	2.7<br>Uniform<br>(1.8,3.6)	--	5.1<br>Uniform<br>(3.3,6.9)	2.3<br>Uniform<br>(1.5,3.1)	2.9<br>Uniform<br>(1.9,3.9)	0.6<br>Uniform<br>(0.4,0.8)
Transportation	2.3<br>Uniform<br>(1.5,3.1)	2.7<br>Uniform<br>(1.8,3.6)	1.5<br>Uniform<br>(1.0,2.0)	7.4<br>Uniform<br>(4.8,10.0)	8.3<br>Uniform<br>(5.4,11.2)	16.5<br>Uniform<br>(10.9,22.2)	0.6<br>Uniform<br>(0.4,0.8)
Domestic	0.6<br>Uniform<br>(0.4,0.8)	0.7<br>Uniform<br>(0.5,0.9)	0.5<br>Uniform<br>(0.3,0.7)	5.1<br>Uniform<br>(3.3,6.9)	0.8<br>Uniform<br>(0.5,1.1)	1.0<br>Uniform<br>(0.7,1.3)	0.4<br>Uniform<br>(0.3,0.5)
Other	1.1<br>Uniform	1.4<br>Uniform	0.9<br>Uniform	5.1<br>Uniform	1.4<br>Uniform	1.8<br>Uniform	0.4<br>Uniform

	(0.7,1.5)	(0.9,1.9)	(0.6,1.1)	(3.3,6.9)	(0.9,1.9)	(1.2,2.4)	(0.3,0.5)

[a] Units: 10^{-3} kg m^{-3}.

Supplementary Table 21 N content of artificial synthesis products

**Type**	**N content (%)** P_{asp}^{CT}
Detergent	0.5 Normal (0.5,0.01)
Drugs	5 Normal (5,1)
Plastic	0.5 Normal (0.5,0.01)
Synthetic fiber	10 Normal (10,4)
Rubber	0.5 Normal (0.5,0.01)
Chemical pesticide	5 Normal (5,1)
Nitrate	22 Normal (22, 19.36)
Synthetic dyes	15 Normal (15, 9)
Industrial explosives	12.5 Normal (12.5, 6.25)

Supplementary Table 22. Comparison with N flow analysis carried out by other studies

N flows and socioeconomy	1995-1996			2004			2012-2015		
	Beijing[63] (1996)	CAP[64] (1995)	Guangzhou (1995)	Shanghai[65] (2004)	Beijing[63] (2004)	Guangzhou (2004)	Beijing[63] (2012)	China[66] (2014)	Guangzhou (2015)
N creation, Gg N y^{-1}									
HBNF	--	--	56.8	~177.7	--	74.5	--	4.7×10^4	85.2
Fossil fuel burning	137.4	33.6	26.0	207.7	151.6	62.3	223.5	1.7×10^4	89.0
Anthropogenic Nr creation ratio	--	88%	90.7%	96.9%	--	94.0%	--	68.6%	95.0%
Nr loss or accumulation, Gg N y^{-1}									
To atmosphere									
NH_3	81.7	8.0	30.5	56.0	57.7	27.7	48.8	--	22.6
NOx	137.4	35.6	34.2	245.4	151.6	65.8	223.5	--	92.3
N_2O	--	4.6	1.4	--	--	1.7	--	2.1×10^3	2.0
To surface water	52.8	1.2	8.1	153.2	64.9	16.4	45.9	1.6×10^4	22.8
To groundwater	--	12.2	10.0	15.9	--	10.1	--	--	11.4
Socioeconomic factors									
Area, thousand km^2	16.4	12.4	7.4	6.3	16.4	7.4	16.4	9.6×10^3	7.4
Population, million	12.6	2.5	10.4	18.3	14.9	9.7	20.7	1.2×10^3	13.5

Supplementary Method

Supplementary Method 1 Coupled human-natural urban nitrogen flow analysis model

Substance flow analysis (SFA) is an important method that quantifies the nitrogen flows throughout the nitrogen cycle process[67], and it paves the way to a transition toward sustainable Nr management[66]. In addition, the uncertainty associated with the equalities and datasets of flow quantification should be noted.[68] Based on SFA, scholars have attempted to develop statistical models to simulate the nitrogen cycling process. The central idea aims to estimate nitrogen fluxes by integrating the measured nitrogen flux data and the quantified impacts with social, economic, and environmental factors[69]. The statistical models often combine the mass balance model with the coefficient model. The former estimates nitrogen accumulation or loss by referring to the input and output of the system[70]. The latter is a semi-empirical model, which can allocate and adjust each nitrogen flux according to the geography, climate, landscape, and other factors[71]. Most statistical models have focused on farmland or natural ecosystems.[72-7374] Recent research has aimed to integrate the role of human activity into a coupled human-natural system in order to study the nitrogen cycling process on a national scale. [66,20] Here, we developed human-natural urban nitrogen flow analysis model comprising two interrelated models, namely, the Full Nitrogen Flow Analysis (FNFA) model and the Nitrogen Network Calculator (NNC).

Supplementary Method 2 Full Nitrogen Flow Analysis (FNFA) model

Based on substance flow analysis (SFA), the Full Nitrogen Flow Analysis (FNFA) model was established to characterize the dynamics of N fate and flux across subsystems throughout the system. Both natural and anthropogenic activities were taken account to, and all Nr emissions sources and interactions among 12 subsystems (farmland, urban green, livestock, forest, aquaculture, industry, human, sewage disposal, garbage disposal, surface water, groundwater and atmosphere) were integrated. The detailed model framework is shown in Supplementary Figure 1.

Each N flow can be quantified either as an input flow into the sink subsystem or as an output flow from the source subsystem. Specific mathematical methods of all N flow can be divided into three categories, first, direct calculation, with N flow calculated by multiplying activity data and related parameters; second, indirect calculation, relying on other flow results to get N flow; third, systemic balance and crosscheck, with unknown or possible flows to be considered by balancing all the known flows of the system and crosschecking with statistical data. Specific calculations were programed in R language and realized with R software and Microsoft Excel, in the sequential order of direct calculation, indirect calculation and systemic balance and crosscheck. Detailed equations in each subsystem and systemic balance were shown below (eq. S1-eq. S26), and the calculation of each input, output and accumulation were described in Supplementary Tables 1-12. Furthermore, the activity data and parameters information in each input, output and

accumulation were interpreted in Supplementary Tables 13- 21. In all subsystems, both the input and output portions can be divided into two categories: created and recycled Nr. Created Nr is produced by human (e.g. HBNF, ABNF, fossil fuel combustion) and natural activities (e.g. NBNF). Recycled Nr is already in the system, and is transported and transformed among the different subsystems (e.g. leaching and runoff).

Farmland

The farmland is the part of terrestrial land which is used for arable farming. Inputs to farmland subsystem include N deposition (I_{dp}^{FL}), ABNF (I_{BNF}^{FL}), chemical N fertilizer (I_{cf}^{FL}), excretion (I_{he}^{FL} by human and I_{le}^{FL} by livestock), irrigation water (I_{ir}^{FL}) and straws back to farmland (I_{st}^{FL}). Among the inputs, ABNF (I_{BNF}^{FL}) is anthropogenic created Nr input to the whole system. Outputs include harvest crops (O_{cp}^{FL}), straws (O_{st}^{FL}) and Nr loss to environment, divided into atmosphere (N_2 ($O_{N_2}^{FL}$), N_2O ($O_{N_2O}^{FL}$), NO (O_{NO}^{FL}), NH_3 ($O_{NH_3}^{FL}$) and NOx ($O_{NO_x}^{FL}$)) and water (surface water (O_{run}^{FL}) and groundwater (O_{lea}^{FL})). Accumulation in farmland(AC^{FL}) is the Nr storage in soil. The calculation equation is shown in eq. S1.

$$AC^{FL} = I_{dp}^{FL} + I_{BNF}^{FL} + I_{cf}^{FL} + I_{he}^{FL} + I_{le}^{FL} + I_{ir}^{FL} + I_{st}^{FL} - O_{cp}^{FL} - O_{st}^{FL} - O_{run}^{FL} - O_{lea}^{FL} - O_{N_2}^{FL} - O_{N_2O}^{FL} - O_{NO}^{FL} - O_{NH_3}^{FL} - O_{NO_x}^{FL} \quad (S1)$$

Livestock

The livestock in city mainly refers to the non-grazing animals, not including the grazing animals. Inputs mainly from the feed N, including straws (I_{st}^{LS}), crops (I_{cp}^{LS}) and urea input for straw ammoniation (I_{cf}^{LS}). Outputs includes livestock products (food (O_{lf}^{LS}) and materials (O_{lm}^{LS})) and Nr loss to the environment (NH_3 volatilization from livestock

excretion ($O_{NH_3}^{LS}$), runoff (O_{run}^{LS}) and leaching (O_{lea}^{LS})). And the accumulation (AC^{LS}) refers to the Nr storage of livestock on hand.

$$AC^{LS} = I_{st}^{LS} + I_{cp}^{LS} + I_{cf}^{LS} - O_{lf}^{LS} - O_{lm}^{LS} - O_{NH_3}^{LS} - O_{run}^{LS} - O_{lea}^{LS} \quad (S2)$$

Forest

The forest in Guangzhou is mainly concentrated in evergreen broadleaf. N inputs to forest subsystem include N deposition (I_{dp}^{FR}) and BNF (I_{BNF}^{FR}), and the outputs include wood material (O_{ft}^{FR}) and Nr loss (N_2O ($O_{N_2O}^{FR}$), N_2 ($O_{N_2}^{FR}$), runoff (O_{run}^{FR}) and leaching (O_{lea}^{FR})) to the environment.

$$AC^{FR} = I_{dp}^{FR} + I_{BNF}^{FR} - O_{ft}^{FR} - O_{run}^{FR} - O_{N_2O}^{FR} - O_{N_2}^{FR} - O_{lea}^{FR} \quad (S3)$$

Industry

The N inputs to industry subsystem are mainly from HBNF (I_{HBNF}^{ID}), fossil fuel burning(I_{ff}^{ID}), and organic materials from other subsystems, including straw transferred to industry for material production (I_{st}^{ID}), crop products transferred to industry(I_{cp}^{ID}), livestock product transferred to industry for material production(I_{lm}^{ID}) and timber from forest used for products (I_{ft}^{ID}). Among the inputs, fossil fuel combustion(I_{ff}^{ID}) and HBNF (I_{HBNF}^{ID}) are anthropogenic created Nr input to the whole system. The outputs refer to the chemical N fertilizer(O_{fer}^{ID}), industrial products (organic(O_{idp-og}^{ID}) and synthetic($O_{idp-asp}^{ID}$) products), NOx from fossil fuel burning(O_{NOx}^{ID}), industrial wastewater (O_{ww}^{ID}), and the denitrification (N_2 ($O_{N_2}^{ID}$) and N_2O ($O_{N_2O}^{ID}$)) during the wastewater treatment. Because the

industry subsystem undertakes production process, no accumulation in the subsystem is assumed.

$$IN^{ID} = I_{HBNF}^{ID} + I_{st}^{ID} + I_{cp}^{ID} + I_{lm}^{ID} + I_{ft}^{ID} + I_{ff}^{ID} \tag{S4}$$

$$OUT^{ID} = O_{fer}^{ID} + O_{idp-og}^{ID} + O_{idp-asp}^{ID} + O_{ww}^{ID} + O_{NOx}^{ID} + O_{N_2}^{ID} + O_{N_2O}^{ID} \tag{S5}$$

Aquaculture

Aquaculture refers to the cultivated fishery. Inputs to aquaculture subsystem are mainly from feed (I_{cp}^{AQ}), N deposition (I_{dp}^{AQ}), Besides the aquaculture products harvest (O_{ap}^{AQ}), Nr outputs to environment through denitrification (N_2 ($O_{N_2}^{AQ}$), N_2O ($O_{N_2O}^{AQ}$)), NH_3 volatilization ($O_{NH_3}^{AQ}$) and runoff (O_{run}^{AQ}).

$$AC^{AQ} = I_{dp}^{AQ} + I_{cp}^{AQ} - O_{ap}^{AQ} - O_{N_2}^{AQ} - O_{N_2O}^{AQ} - O_{NH_3}^{AQ} - O_{run}^{AQ} \tag{S6}$$

Human

The human subsystem is the core consumption process connecting the production and treatment process. The agricultural food (crop (I_{cp}^{HM}), livestock (I_{lf}^{HM}) and aquaculture (I_{ap}^{HM})), industrial products (organic (I_{idp-og}^{HM}) and synthetic ($I_{idp-asp}^{HM}$)), and energy (fossil fuel (I_{ff}^{HM}) and biomass energy (I_{stbu}^{HM})) are considered as the inputs to the human subsystem. Among the inputs, fossil fuel combustion(I_{ff}^{HM}) is anthropogenic created Nr input to the whole system. After human consumption, the food inputs are accumulated in human organism, or discharge as excretion (O_{he}^{HM}), NH_3 volatilization ($O_{NH_3}^{HM}$) and wastewater (treated (O_{tds}^{HM}) and untreated (O_{utds}^{HM})). For the industrial products, partial products accumulated in human settlements, and the rest is sent to disposal as garbage (O_{gb}^{HM}).

During the energy consumption, NOx ($O_{NO_x}^{HM}$) would be released to the air.

$$AC^{HM} = I_{cp}^{HM} + I_{lf}^{HM} + I_{ap}^{HM} + I_{idp-og}^{HM} + I_{idp-asp}^{HM} + I_{ff}^{HM} + I_{stbu}^{HM} - O_{tds}^{HM} - O_{utds}^{HM} - O_{he}^{HM} - O_{NO_x}^{HM} - O_{NH_3}^{HM} - O_{gb}^{HM} \quad (S7)$$

Urban green

The urban green mainly refers to urban lawn and horticultural shrub. The inputs are from N deposition (I_{dp}^{UG}) and BNF (I_{BNF-ug}^{UG}), and N outputs to the environment as gaseous (denitrification N_2 ($O_{N_2}^{UG}$), denitrification N_2O ($O_{N_2O}^{UG}$), NO (O_{NO}^{UG}) and NH_3 ($O_{NH_3}^{UG}$)) and liquid loss (runoff (O_{run}^{UG}) and leaching (O_{lea}^{UG})).

$$AC^{UG} = I_{dp}^{UG} + I_{BNF-ug}^{UG} - O_{run}^{UG} - O_{lea}^{UG} - O_{N_2}^{UG} - O_{N_2O}^{UG} - O_{NO}^{UG} - O_{NH_3}^{UG} \quad (S8)$$

Sewage disposal

Sewage disposal is the N removing subsystem that treats liquid waste to reduce its environmental impacts. The sewage sent to the disposal facilities (I_{ds}^{SD}) is as inputs, and the outputs are discharged as sludge (O_{ss}^{SD}), treatment effluent (O_{ste}^{SD}), N_2 ($O_{N_2}^{SD}$) and N_2O ($O_{N_2O}^{SD}$) through denitrification process. No accumulation is assumed in the sewage disposal subsystem.

$$IN^{SD} = I_{ds}^{SD} \quad (S9)$$

$$OUT^{SD} = O_{N_2}^{SD} + O_{N_2O}^{SD} + O_{ss}^{SD} + O_{ste}^{SD} \quad (S10)$$

Garbage disposal

Garbage disposal is the N removing subsystem that treats solid waste to reduce its

environmental impacts. The garbage is mainly from human consumption (I_{hg}^{GD}) and sewage sludge (I_{ss}^{GD}). And outputs include the garbage sent to landfill (O_{gdl}^{GD}), composting (O_{gdc}^{GD}) and recycling (O_{gut}^{GD}). Partial untreated garbage (O_{got}^{GD}) is directly released to environment. No accumulation is considered in this subsystem.

$$IN^{GD} = I_{hg}^{GD} + I_{ss}^{GD} \tag{S11}$$

$$OUT^{GD} = O_{gut}^{GD} + O_{gdl}^{GD} + O_{gdc}^{GD} + O_{got}^{GD} \tag{S12}$$

Surface water

Surface water inputs Nr from other subsystems, including N deposition (I_{dp}^{SW}), runoff from farmland (O_{run}^{FL}), livestock (O_{run}^{LS}), forest (O_{run}^{FR}), aquaculture (O_{run}^{AQ}) and urban green (O_{run}^{UG}), industrial wastewater (O_{ww}^{ID}), untreated domestic sewage (O_{utds}^{HM}) and sewage treatment effluent (O_{ste}^{SD}). Nr outputs through denitrification(N_2 ($O_{N_2}^{SW}$) and N_2O ($O_{N_2O}^{SW}$)) and irrigation water (O_{ir}^{SW}). The difference between inputs and outputs is considered as Nr transfer to oceans ($Trans^{OC}$).

$$IN^{SW} = I_{dp}^{SW} + O_{run}^{FL} + O_{run}^{FR} + O_{run}^{LS} + O_{ww}^{ID} + O_{run}^{AQ} + O_{utds}^{HM} + O_{run}^{UG} + O_{ste}^{SD} \tag{S13}$$

$$OUT^{SW} = O_{ir}^{SW} + O_{N_2}^{SW} + O_{N_2O}^{SW} \tag{S14}$$

$$Trans^{OC} = IN^{SW} - OUT^{SW} \tag{S15}$$

Groundwater

Groundwater subsystem receives Nr from other subsystems, including leaching in farmland (O_{lea}^{FL}), livestock (O_{lea}^{LS}), forest (O_{lea}^{FR}) and urban green (O_{lea}^{UG}). No N outputs from this subsystem is assumed for the short term, and all the input Nr accumulates in this subsystem(AC^{GW}).

$$AC^{GW} = IN^{GW} = O_{lea}^{FL} + O_{lea}^{LS} + O_{lea}^{FR} + O_{lea}^{UG} \tag{S16}$$

Atmosphere

Atmosphere subsystem inputs N (N_2, N_2O, NOx and HN_3) from other subsystems. Denitrification (N_2 and N_2O) are mainly from farmland ($O_{N_2}^{FL}$ and $O_{N_2O}^{FL}$), forest ($O_{N_2}^{FR}$ and $O_{N_2O}^{FR}$), industry ($O_{N_2}^{ID}$ and $O_{N_2O}^{ID}$), aquaculture ($O_{N_2}^{AQ}$ and $O_{N_2O}^{AQ}$), urban green ($O_{N_2}^{UG}$ and $O_{N_2O}^{UG}$), sewage disposal ($O_{N_2}^{SD}$ and $O_{N_2O}^{SD}$) and surface water ($O_{N_2}^{SW}$ and $O_{N_2O}^{SW}$). NOx emissions are from industrial fossil fuel combustion (O_{NOx}^{ID}), human fossil fuel combustion ($O_{NO_x}^{HM}$), straw burning ($O_{NO_x}^{FL}$) and NO emission $\sum O_{NO}$. NH_3 volatilization are from farmland $O_{NH_3}^{FL}$, livestock ($O_{NH_3}^{LS}$), aquaculture ($O_{NH_3}^{AQ}$), human ($O_{NH_3}^{HM}$) and urban green ($O_{NH_3}^{UG}$). Nr outputs through deposition (O_{dp}^{AT}). The difference between inputs Nr and outputs Nr is considered as Nr transfer to surrounding areas ($Trans^{AT}$).

$$IN_{N_2}^{AT} = O_{N_2}^{FL} + O_{N_2}^{FR} + O_{N_2}^{ID} + O_{N_2}^{AQ} + O_{N_2}^{UG} + O_{N_2}^{SD} + O_{N_2}^{SW} \tag{S17}$$

$$IN_{N_2O}^{AT} = O_{N_2O}^{FL} + O_{N_2O}^{FR} + O_{N_2O}^{ID} + O_{N_2O}^{AQ} + O_{N_2O}^{UG} + O_{N_2O}^{SD} + O_{N_2O}^{SW} \tag{S18}$$

$$IN_{NOx}^{AT} = O_{NO_x}^{FL} + O_{NOx}^{ID} + O_{NO_x}^{HM} + \sum O_{NO} \tag{S19}$$

$$IN_{NH_3}^{AT} = O_{NH_3}^{FL} + O_{NH_3}^{LS} + O_{NH_3}^{AQ} + O_{NH_3}^{HM} + O_{NH_3}^{UG} \tag{S20}$$

$$OUT^{AT} = O_{dp}^{AT} \tag{S21}$$

$$Trans^{AT} = IN_{N_2O}^{AT} + IN_{NOx}^{AT} + IN_{NH_3}^{AT} - OUT^{AT} \tag{S22}$$

Balance and crosscheck

To balance the food and industrial products production and consumption driven by

human, the crop, livestock and aquaculture food and industrial products import (IM^{cp}, IM^{lf}, IM^{ap} and IM^{ip}), export (EX^{cp}, EX^{lf}, EX^{ap} and EX^{ip}) and net domestic trade (NDT^{cp}, NDT^{lf}, NDT^{ap} and NDT^{ip}) are taken into consideration. And the N flows through DPS are calculated by balancing all the other known flows and crosschecked with corresponding statistical data specifically.

$$IM^{cp} + O_{cp}^{FL} = I_{cp}^{HM} + I_{cp}^{LS} + I_{cp}^{LS} + EX^{cp} + NDT^{cp} \quad \text{(S23)}$$

$$IM^{lf} + O_{lf}^{LS} = I_{lf}^{HM} + EX^{lf} + NDT^{lf} \quad \text{(S24)}$$

$$IM^{ap} + O_{ap}^{AQ} = I_{lf}^{HM} + EX^{ap} + NDT^{ap} \quad \text{(S25)}$$

$$IM^{ip} + I_{HBNF}^{ID} = O_{fer}^{ID} + I_{idp-asp}^{HM} + EX^{ip} + NDT^{ip} \quad \text{(S26)}$$

Supplementary Method 3 Nitrogen network calculator(NNC) model

To help to organize and synthesize massive information during the N balance calculation, the NNC was proposed. The model comprises an independent data part and a model part (Supplementary Figure 2). For the data part (Supplementary Tables 13-21), all variables (i.e., activity data, such as fertilizer production, and literature-based parameters, such as deposition coefficient) were collected and attributed continuous distributions. For the model part (Supplementary Tables 1-12), all input, output, and accumulation quantitative equations were formulated and collected. The nitrogen balances with uncertainty in all subsystems were simultaneously quantified by interactively calling data part and model part with R software. Based on the NNC, the N balance calculation data and equations were collected, stored, and shared in a unified pattern. The independence between the data part and the model part facilitated modifications and improved efficiency.

Supplementary Method 4 Uncertainty Analysis

Although our calculations were based on all available information, we note that there were uncertainties in respect to certain aspects of quantification models and datasets. The FNFA and NNC models that we used to estimate fluxes composed a tightly interwoven system, such that the alteration of one variable, input, or output caused a ripple effect through other subsystems and impact the whole system.

We conducted the Monte Carlo simulation to test the propagation of input uncertainties into the successive and final results. The variables in the data part (including activity data and parameters) were attributed continuous distributions instead of a single point estimate. For activity data, uniform distribution was applied and different coefficients of variation(CVs) were set according to the statistical source of the data (Supplementary Table 12). Activity data obtained by the technical measurement (e.g. forest area) or manual investigation (e.g. crop production) were provided with uniform distribution with CV values of 0.1, obtained by calculation based on primary source (e.g. disposal rate of domestic wastewater) were provided with uniform distribution with CV values of 0.2. For parameters, continuous distributions (triangular, uniform or normal) were provided and different coefficient of CVs were given by considering data quality (Supplementary Tables 13-21). For those parameters which can be consulted for more than one reference, the triangular distributions were assumed; while for parameters from one single reference, normal distribution and uniform distributions were applied and CVs were set according to

the qualitative assessment of their data qualities. We classified the CVs of parameter into three grades: high, moderate, and low reliabilities, which were assumed to have the values of 0.1, 0.2, and 0.3, respectively.[75]

The simulation model ran 10,000 trials by randomly selecting values from the input distributions to generate ranges of outcomes. Besides the single-point estimates, uncertainty analysis of the N flows including means, SDs, CVs, 5th and 95th percentiles were provided. Partial N flow simulation results with uncertainty are provided in Supplementary Figure 15. Moreover, we compared the partial N flows with previous estimates from China and other cities, which is provided in the Supplementary Table 22.

Supplementary Method 5 STIRPAT model

A better understanding the human impact on nitrogen creation can provide a basis for N management strategies that can develop a more sustainable N cycle. The Stochastic Impacts by Regression on Population, Affluence, and Technology (STIRPAT) model is reformulated from the IPAT (Impact = Population ·Affluence ·Technology) model, developed by Dietz and Rosa[76]. STIRPAT is a statistical model that is used to assess the effects of human activities on the environment, and it has been used successfully to estimate the impact of anthropogenic factors on greenhouse gas (GHG) and nitrogen emissions[47,77,78].

The STIRPAT model was used to assess the contributions of socioeconomic factors to changes in anthropogenic Nr creation. The standard STIRPAT model (eq. S26) is:

$$I = aP^{b}A^{c}T^{d}e \tag{S26}$$

As a nonlinear multivariate equation of the standard STIRPAT model, it is difficult to calculate the values of a, b, c, d, and e. All of the variables in eq.S26 are often taken logarithms to facilitate the calculation[77,79]:

$$\ln I = a + b\ln P + c\ln A + d\ln T + e \tag{S27}$$

where, P, A, and T represent population, affluence, and technology. b, c, and d are the coefficients of P, A, and T, respectively; a is the constant, and e is the error term.

Here, the extended STIRPAT model was used to assess the contributions of socioeconomic factors (i.e., population, dietary choice, energy intensity, and industrial level) to changes in anthropogenic Nr creation (eq. S28), and ordinary least squares (OLS)

regression was used to evaluate the variables.

$$\ln I = a + b\ln P + c\ln A + d_1 \ln T_1 + d_2 \ln T_2 + e \tag{S28}$$

where, P, A, T_1, and T_2 represent population, dietary choice, energy intensity and industrial level. b, c, d_1, and d_2 are the coefficients of P, A, T_1, and T_2, respectively; a is the constant, and e is the error term.

Supplementary Discussions

Supplementary Discussion 1 Human drivers to Nr creation changes analysis

We tested the influence of six related socioeconomic factors (population, diet choice, GDP per capital, energy intensity, industrial level and industrial structure) on anthropogenic Nr creation and also the correlation among all the factors through the Pearson correlation method (Supplementary Figure 16a). Finally, four sociological factors: population, diet choice, energy intensity and industrial level were selected. The OLS regression estimate of the extended STIRPAT model was used to examine the contributions of four selected individual factors to anthropogenic Nr creation. The regression coefficients of all explanatory variables were significant and the R square was 0.940, indicating a good reliability for goodness-of-fit (Supplementary Figure 16b).

Supplementary Discussion 2 Comparison with N flow analysis carried out by other studies

We compared the results of our study with previous estimates from other cities and the whole of China for the same period (Supplementary Table 22). Among them, Beijing and Shanghai are typical international metropolises in China, and the Central Arizona-Phoenix(CAP) represents the watershed which includes the Phoenix metropolitan area and the surrounding agricultural land and desert in the United States.[64] This study aimed to investigate the level of Nr creation and the associated environmental effects in the human-dominated urban system. To examine this issue, N creation and loss or accumulation in the environment were selected for comparison with other studies.

The calculation of HBNF in Shanghai[65] was much higher than that in Guangzhou in 2004. Both of these studies accounted of nitrogen fertilizer and synthetic ammonia production in the HBNF budget. More developed and large-scale manufacturing fixed more nitrogen into human production and consumption in Shanghai. Our estimate of N fixation through fossil fuel burning was lower than the estimates for Shanghai and Beijing, whereas similar to that outlined in CAP during the same period. In fact, Shanghai consumed more fossil fuels than Beijing and Guangzhou. In 2015, the total energy consumption of Shanghai was 113.9 million tons of SCE, whereas total energy consumption was 5.7 million tons in Guangzhou and 6.9 million tons in Beijing, respectively. NOx emissions in Beijing were estimated by using higher emission factors of different fuel types[39,80]. The

anthropogenic Nr creation ratios in cities were all much higher than the average level for all of China, which also indicated that human activities in urban areas have greatly accelerated Nr creation.

Estimates of NH_3 emissions in Beijing and Guangzhou declined as the cities developed, and this finding was consistent with the reduction in farmland area and the reduction in ammonium-based fertilizers. In contrast to the results for all of China, which revealed a minimum ratio of 2:1 in respect to NH_3-N/NO_x-N by large-scale monitoring of N deposition,[81] the emission ratios of NH_3 to NOx in Beijing, Shanghai, and Guangzhou were much smaller. NOx emissions in cities were higher than NH_3 emissions, which is consistent with NO_2 pollution in the urban atmosphere.[82] Higher levels of Nr to surface water were estimated in Beijing and Guangzhou, which was attributed to a dense population, production, and living discharge. Furthermore, the value in Shanghai and Beijing reflected all Nr inputs into surface water rather than the difference between the Nr inputs and outputs (e.g., denitrification process, irrigation water to farmland), and the Nr accumulation in groundwater among different cities were comparative.

Overall, the estimates of Nr flow in Guangzhou matched previous studies of other cities that considered the city scales, populations, and the intensity of economic activity. Furthermore, in human-dominated urban systems, Nr creation and the associated environmental effects were more dramatic than the average level for all of China during the same period.

Supplementary References

1. Guangzhou Municipal Statistics Bureau. *Guangzhou Statistical Yearbook*. (National Bureau of Statistics of China Press, 2016).
2. Editorial Board of the Guangdong Statistical Yearbook on Agriculture. *Guangdong Statistical Yearbook on Agriculture*. (National Bureau of Statistics of China Press).
3. Guangzhou Bureau of Environmental Protection. *Guangzhou Environmental Quality Report*.
4. National Bureau of Statistic. *China City Statistical Yearbook*. (National Bureau of Statistics of China Press).
5. National Bureau of Statistic. *China Statistical Yearbook on Environment*. (China Environment Yearbook Press).
6. Yan, W., Yin, C. & Zhang, S. Nutrient budgets and biogeochemistry in an experimental agricultural watershed in Southeastern China. *Biogeochemistry* **45,** 1–19 (1999).
7. Herridge, D. F., Peoples, M. B. & Boddey, R. M. Global inputs of biological nitrogen fixation in agricultural systems. *Plant Soil* **311,** 1–18 (2008).
8. Xing, G. X. & Zhu, Z. L. Regional nitrogen budgets for China and its major watersheds. *Biogeochemistry* **57/58,** 405–427 (2002).

9. Xie, G., Wang, X., Han, D. & Xue, S. Harvest index and residue factor of non-cereal crops in China(in Chinese). *J. China Agric. Univ.* **16,** 9–17 (2011).

10. Zhang, F. Harvest index for various crops in China(in Chinese). *Sci. Agric. Sin.* **2,** 83–87 (1990).

11. Liu, H., Jiang, G. M., Zhuang, H. Y. & Wang, K. J. Distribution, utilization structure and potential of biomass resources in rural China: With special references of crop residues. *Renew. Sustain. Energy Rev.* **12,** 1402–1418 (2008).

12. Gao, X., Wuhan & Beijing. Analysis on the Current Status of Utilization of Crop Straw in China(in Chinese). *J. Huazhong Agric.Univ* **354,** 4031–4036 (2002).

13. Gao *et al.* Estimation of nutrient resource quantity of crop straw and its utilization situation in China(in Chinese). *Trans. Chinese Soc. Agric. Eng.* **25,** 173–179 (2009).

14. Liu, X. . Nitrogen Cycling and Balance in "Agriculture-Livestock-Nutrition-Environment" System of China(in Chinese). (Agricultural University of Hebei, 2005).

15. Ti, C., Pan, J., Xia, Y. & Yan, X. A nitrogen budget of mainland China with spatial and temporal variation. *Biogeochemistry* **108,** 381–394 (2012).

16. Ju, X. *et al.* Reducing environmental risk by improving N management in intensive Chinese agricultural systems. *Proc. Natl. Acad. Sci.* **106,** 3041–3046 (2009).

17. Xing, G. X. & Zhu, Z. L. An assessment of N loss from agricultural fields to the environment in China. *Nutr Cyc Agroecosys* **57,** 67–73 (2000).

18. Zhou, F. *et al.* A New High-Resolution N2O Emission Inventory for China in 2008. *Environ. Sci. Technol.* **48,** 8538–8547 (2014).

19. Xu, Z. *et al.* Nitrogen Balance in a Highly Fertilized Rice–Wheat Double-Cropping System in Southern China. *Soil Sci. Soc. Am. J.* **76,** 1068 (2012).

20. Gu, B., Ju, X., Chang, J., Ge, Y. & Vitousek, P. M. Integrated reactive nitrogen budgets and future trends in China. *Proc. Natl. Acad. Sci.* **112,** 8792–8797 (2015).

21. Yang, Y. *China Food composition list*. (Beijing Medical University Press, 2005).

22. Xie, X. M. *et al.* Study on the Development and Utilization of Different Leather Proteins(in Chinese). *J. Inn. Mong. Univ. Natl.* **19,** 186–189 (2004).

23. Xing, G. X. & Yan, X. Y. Direct nitrous oxide emissions from agricultural fields in China estimated by the revised 1996 IPPC guidelines for national greenhouse gases. *Environ. Sci. Policy* **2,** 355–361 (1999).

24. Li, S., Liu, R. & Shan, H. Nutrient Contents in Main Animal Manures in China(in Chinese). *J. Agro-Environ. Sci.* **28,** 179–184 (2009).

25. Wang, F., Ma, W., Dou, Z. & Ma, L. The estimation of the production amount of animal manure and its environmental effect in China(in Chinese). *China Environ. Sci.* **26,** 614–617 (2006).

26. Yang, C. . *Pollution Situation Investigation and Prevention and Cure*

Countermeasure of National Scale Breeding of Livestock and Poultry. *China Environmental Science Press* (2002).

27. Cai, B., Liu, C. & Chen, C. *City's Greenhouse Gas (GHG) Emission Inventory Research(in Chinese)*. (Chemical Industry Press, 2009).

28. Liu, X. L., Jun-Xiang, X. U., Wang, F. H., Zhang, F. S. & Wen-Qi, M. A. The resource and distribution of nitrogen nutrient in animal excretion in China(in Chinese). *J. Agric. Univ. Hebei* **5,** 27–32 (2005).

29. Sun, Q. & Wang, M. Ammonia Emission and Concentration in the Atmosphere over China(in Chinese). *Sci. Atmos. Sin.* **21,** 590–598 (1997).

30. Huang, H. X. *et al.* Analysis on the status of organic fertilizer and its development strategies in China. *Soil Fertil. Sci. China* **1,** 1–8 (2006).

31. Gu, B. *et al.* The role of industrial nitrogen in the global nitrogen biogeochemical cycle. *Sci. Rep.* **3,** 2579 (2013).

32. Xi, J., Zhang, F. & You, X. Nitrogen balance of natural forest ecosystem in China. *Acta. Ecol. Sin.* **27,** 3257–3267 (2007).

33. Son, Y. Non-symbiotic nitrogen fixation in forest ecosystems. *Ecol. Res.* **16,** 183–196 (2010).

34. Xiong, M. & Bao, F. Study on Balance between Supply and Demand for Timber and Timber Products in China(in Chinese). *Chinese For. Sci. Technol.* **48,** 84–91 (2005).

35. Wu, S. Present situation of comprehensive utilization of agricultural straws in China(in Chinese). *China Wood-based Panels* **12,** 1–4 (2005).

36. Boring, L. R., Swank, W. T., Waide, J. B. & Henderson, G. S. Sources, fates, and impacts of nitrogen inputs to terrestrial ecosystems: review and synthesis. *Biogeochemistry* **6,** 119–159 (1988).

37. Fang, Y. *et al.* Atmospheric deposition and leaching of nitrogen in Chinese forest ecosystems. *J. For. Res.* **16,** 341–350 (2011).

38. Fang, Y., Gundersen, P., Mo, J. & Zhu, W. Nitrogen leaching in response to increased nitrogen inputs in subtropical monsoon forests in southern China. *For. Ecol. Manag.* **257,** 332–342 (2009).

39. Gu, B. *et al.* Atmospheric reactive nitrogen in china: Sources, recent trends, and damage costs. *Environ. Sci. Technol.* **46,** 9420–9427 (2012).

40. Liu, H. A review: nutrient requirement of aquatic animals cultivated in China. *J. Dalian Fish. Univ.* **17,** 187–195 (2002).

41. Zhang, X. P. Effect of pollution source at sea on environmental quality in the sea area of Xiamen. *Mar. Environ. Sci.* **20,** 38–41 (2001).

42. Yang, Z. Y. *et al.* Study on the total nitrogen pollution and its environmental costs in aquaculture industry-A positive study on water conversation area of Dianshanhu lake. *Ecol. Econ.* **10,** 83–87 (2007).

43. Chen, J., Hu, G., Qu, J. & Fan, E. TN and TP from pond crab farming in the Taihu

Valley. *Rural Eco-environment* **21,** 21–23 (2005).

44. Zhang, Y. Z. *et al.* Discussion on Estimating Nitrogen and Phosphorus Pollution Loads in Aquaculture(in Chinese). *Jounal Xiamen Univ.* **42,** 223–227 (2003).

45. Shu, T. F., Wen, Y. M. & Tang, Y. T. Cycle and Budget Balance of Nitrogen in the Cultivated Water(in Chinese). *Fish. Sci.* **21,** 30–34 (2002).

46. Zhai, F. Y. *et al.* Study on the current status and trend of food consumption among Chinese population. *Chinese J. Epidemiol.* **26,** 485–488 (2005).

47. Cui, S., Shi, Y., Groffman, P. M., Schlesinger, W. H. & Zhu, Y.-G. Centennial-scale analysis of the creation and fate of reactive nitrogen in China (1910-2010). *Proc. Natl. Acad. Sci.* **110,** 2052–2057 (2013).

48. Gao, L. Analysis and Evaluation of Nitrogen Flow in the Food Chain System—A Case Study of Huang-Huai-Hai Region(in Chinese). (Agricultural University of Hebei, 2009).

49. Gu, B. *et al.* Anthropogenic Modification of the Nitrogen Cycling within the Greater Hangzhou Area System, China. *Ecol. Appl. A Publ. Ecol. Soc. Am.* **19,** 974–988 (2009).

50. Xu, S., Zhang, J. & Fu, Q. Denitrification and phosphorus removal regulation parameters of municipal sewage treatment plant(in Chinese). *Water Supply Drain.* **134,** 7–11 (2007).

51. Lofton, D. D., Hershey, A. E. & Whalen, S. C. Evaluation of denitrification in an

urban stream receiving wastewater effluent. *Biogeochemistry* **86,** 77–90 (2007).

52. Li, Y., Chen, T., Luo, W., Huang, Q. & Wu, J. Contents of organic matter and major nutrients and the ecological effect related to land application of sewage sludge in China(in Chinese). *Acta Ecol. Sin.* **23,** 2464–2474 (2003).

53. Ma, N., Chen, L. & Xiong, F. Disposal and reuse of city sludge in China(in Chinese). *Ecol. Environ.* **12,** 92–95 (2003).

54. Jin, R. Discussion of sludge treatment of urban wastewater plant in China(In Chinese). *J. Wuhan Urban Constr. Inst.* **11,** 1–12 (1994).

55. Lu, Y., Wu, X. & Guo, J. Characteristics of municipal solid waste and sewage sludge co-composting. *Waste Manag.* **29,** 1152–1157 (2009).

56. Wang, H. & Nie, Y. Municipal Solid Waste Characteristics and Management in China. *J. Air Waste Manag. Assoc.* **51,** 250–263 (2001).

57. Huang, Q., Wang, Q., Dong, L., Xi, B. & Zhou, B. The current situation of solid waste management in China. *J. Mater. Cycles Waste Manag.* **8,** 63–69 (2006).

58. Li, G. & Zhang, F. *Solid waste composting and organic compound fertilizer production*. (Chemical Industry Press, 2000).

59. Lu, C. & Tian, H. Spatial and temporal patterns of nitrogen deposition in China. *J. Geophys. Res.* **112,** D22 (2007).

60. Tang, X. & Wang, S. Trends of the precipitation acidity over China during 1992-2006. *Chin Sci. Bull.* **55,** 1800–1807 (2010).

61. Liu, X. *et al.* Enhanced nitrogen deposition over China. *Nature* **494,** 459–462 (2013).

62. Liu, X. J. & Zhang, F. S. Nutrient from environment and its effect in nutrient resources management of ecosystems: a case study on atmospheric nitrogen deposition. *Arid Zo. Res.* **26,** 306–311 (2009).

63. Zhang, Y., Lu, H., Fath, B. D. & Zheng, H. Modelling urban nitrogen metabolic processes based on ecological network analysis: A case of study in Beijing, China. *Ecol. Modell.* **337,** 29–38 (2016).

64. Baker, L. A., Hope, D., Xu, Y., Edmonds, J. & Lauver, L. Nitrogen balance for the Central Arizona-Phoenix (CAP) ecosystem. *Ecosystems* **4,** 582–602 (2001).

65. Gu, B. *et al.* The long-term impact of urbanization on nitrogen patterns and dynamics in Shanghai, China. *Environ. Pollut.* **171,** 30–37 (2012).

66. Luo, Z., Hu, S., Chen, D. & Zhu, B. From Production to Consumption: A Coupled Human-Environmental Nitrogen Flow Analysis in China. *Environ. Sci. Technol.* **52,** 2025–2035 (2018).

67. Coppens, J., Meers, E., Boon, N., Buysse, J. & Vlaeminck, S. E. Follow the N and P road: High-resolution nutrient flow analysis of the Flanders region as precursor for sustainable resource management. *Resour. Conserv. Recycl.* **115,** 9–21 (2016).

68. Liu, X. *et al.* Intensification of phosphorus cycling in China since the 1600s. *Proc. Natl. Acad. Sci.* **113,** 2609–2614 (2016).

69. Alexander, R. B., Johnes, P. J., Boyer, E. W. & Smith, R. A. A Comparison of Models for Estimating the Riverine Export of Nitrogen from Large Watersheds. *Biogeochemistry* **57/58,** 295–339 (2002).

70. Kaye, J. P., Groffman, P. M., Grimm, N. B., Baker, L. A. & Pouyat, R. V. A distinct urban biogeochemistry? *Trends Ecol. Evol.* **21,** 192–199 (2006).

71. Johnes, P. J. & Heathwaite, A. L. Modelling the impact on water quality of land use change in agricultural catchments. (1997).

72. Fujimaki, R., Sakai, A. & Kaneko, N. Ecological risks in anthropogenic disturbance of nitrogen cycles in natural terrestrial ecosystems. *Ecol. Res.* **24,** 955–964 (2009).

73. Sun B, Shen RP & Bouwman AF. Surface N Balances in Agricultural Crop Production Systems in China for the Period 1980-2015. **18,** (Beijing Jiaotong University, 2008).

74. Wang, J., Pei, Y. S., Zhang, K. J., Gao, G. & Yang, Z. F. Investigating the spatial–temporal variation of nitrogen cycling in an urban river in the North China Plain. *Water Sci. Technol.* **63,** 2553–2559 (2011).

75. Laner, D., Rechberger, H. & Astrup, T. Systematic Evaluation of Uncertainty in Material Flow Analysis. *J. Ind. Ecol.* **18,** 859–870 (2015).

76. Dietz, T. & Rosa, E. A. Rethinking the environmental impacts of population, affluence and technology. *Hum. Ecol. Rev.* **1,** 277–300 (1994).

77. Jiang, S. *et al.* Enhanced nitrogen and phosphorus flows in a mixed land use basin: Drivers and consequences. *J. Clean. Prod.* **181,** 416–425 (2018).

78. Wang, P., Wu, W., Zhu, B. & Wei, Y. Examining the impact factors of energy-related CO2 emissions using the STIRPAT model in Guangdong Province, China. *Appl. Energy* **106,** 65–71 (2013).

79. York, R., Rosa, E. A. & Dietz, T. STIRPAT, IPAT and ImPACT: analytic tools for unpacking the driving forces of environmental impacts. *Ecol. Econ.* **46,** 351–365 (2003).

80. Kato, N. & Akimoto, H. Anthropogenic emissions of SO2 and NOx in Asia: emission inventories. *Atmos. Environ.* **26,** 2997–3017 (2007).

81. Xuejun, L. *et al.* Enhanced nitrogen deposition over China. *Nature* **494,** 459–462 (2013).

82. Environmental Status Bulletin of Guangzhou. (2016). Available at: www.gzepb.gov.cn/.
