# Supplementary material for: Aggravation of reactive nitrogen flow driven by human production and consumption in Guangzhou City China
Source: Nat Commun. 2020 Mar 5;11:1209. doi: 10.1038/s41467-020-14699-x (PMC7058066; doi:10.1038/s41467-020-14699-x)
Supplement: Supplementary file 2 — Reporting Summary [file 41467_2020_14699_MOESM2_ESM.pdf]

nature research

Corresponding author(s): Linyu Xu

Last updated by author(s): Jan 10, 2020

Reporting Summary

Nature Research wishes to improve the reproducibility of the work that we publish. This form provides structure for consistency and transparency in reporting. For further information on Nature Research policies, see Authors & Referees and the Editorial Policy Checklist.

Statistics

For all statistical analyses, confirm that the following items are present in the figure legend, table legend, main text, or Methods section.

n/a	Confirmed	
☐	☒	The exact sample size (n) for each experimental group/condition, given as a discrete number and unit of measurement
☒	☐	A statement on whether measurements were taken from distinct samples or whether the same sample was measured repeatedly
☐	☒	The statistical test(s) used AND whether they are one- or two-sided<br>*Only common tests should be described solely by name; describe more complex techniques in the Methods section.*
☐	☒	A description of all covariates tested
☐	☒	A description of any assumptions or corrections, such as tests of normality and adjustment for multiple comparisons
☐	☒	A full description of the statistical parameters including central tendency (e.g. means) or other basic estimates (e.g. regression coefficient) AND variation (e.g. standard deviation) or associated estimates of uncertainty (e.g. confidence intervals)
☐	☒	For null hypothesis testing, the test statistic (e.g. F, t, r) with confidence intervals, effect sizes, degrees of freedom and P value noted<br>*Give P values as exact values whenever suitable.*
☒	☐	For Bayesian analysis, information on the choice of priors and Markov chain Monte Carlo settings
☒	☐	For hierarchical and complex designs, identification of the appropriate level for tests and full reporting of outcomes
☒	☐	Estimates of effect sizes (e.g. Cohen's d, Pearson's r), indicating how they were calculated

Our web collection on statistics for biologists contains articles on many of the points above.

Software and code

Policy information about availability of computer code

Data collection	The main data sources are as follows: (1) national, provincial, and urban statistical databases; (2) provincial and urban survey and planning documents; and (3) existing research results and methodologies.
Data analysis	We organize and synthesize large amounts of information with uncertainty using R software when carrying out the N balance calculation

For manuscripts utilizing custom algorithms or software that are central to the research but not yet described in published literature, software must be made available to editors/reviewers. We strongly encourage code deposition in a community repository (e.g. GitHub). See the Nature Research guidelines for submitting code & software for further information.

Data

Policy information about availability of data

All manuscripts must include a data availability statement. This statement should provide the following information, where applicable:

- Accession codes, unique identifiers, or web links for publicly available datasets
- A list of figures that have associated raw data
- A description of any restrictions on data availability

All relevant data are publicly available in the supplementary materials and online data repositories, and are available from the authors. The sources of activity data, parameters for N flow analysis are provided in Supplementary Data 1. The source data underlying Figure 2a, 2b, 2c, 4a, 4b, 4c and 4d are provided in Supplementary Data 2.

Field-specific reporting

Please select the one below that is the best fit for your research. If you are not sure, read the appropriate sections before making your selection.

☐ Life sciences ☐ Behavioural & social sciences ☒ Ecological, evolutionary & environmental sciences

For a reference copy of the document with all sections, see nature.com/documents/nr-reporting-summary-flat.pdf

Ecological, evolutionary & environmental sciences study design

All studies must disclose on these points even when the disclosure is negative.

Study description	The primary purposes of this study are to develop a new coupled human-natural nitrogen flow analysis model at the urban scale and then use this model to investigate how the N flow originated, modified, and affected the environment in a typical megalopolis.
Research sample	N/A
Sampling strategy	N/A
Data collection	The main data sources are as follows: (1) national, provincial, and urban statistical databases; (2) provincial and urban survey and planning documents; and (3) existing research results and methodologies.
Timing and spatial scale	Guangzhou China 1995-2015
Data exclusions	N/A
Reproducibility	N flow analysis in our study could be reproduced according to the coupled human-natural urban nitrogen flow analysis model and corresponding data in supplementary materials.
Randomization	We conducted the Monte Carlo simulation to test the propagation of input uncertainties into the successive and final results.
Blinding	We collect data from statistical databases and existing research results.

Did the study involve field work? ☐ Yes ☒ No

Reporting for specific materials, systems and methods

We require information from authors about some types of materials, experimental systems and methods used in many studies. Here, indicate whether each material, system or method listed is relevant to your study. If you are not sure if a list item applies to your research, read the appropriate section before selecting a response.

Materials & experimental systems

n/a	Involved in the study	
☒	☐	Antibodies
☒	☐	Eukaryotic cell lines
☒	☐	Palaeontology
☒	☐	Animals and other organisms
☒	☐	Human research participants
☒	☐	Clinical data

Methods

n/a	Involved in the study	
☒	☐	ChIP-seq
☒	☐	Flow cytometry
☒	☐	MRI-based neuroimaging
